# Effects of solamargine in hepatic metastasis of colorectal cancer: induction of ferroptosis and elimination of cancer stem cells

**DOI:** 10.1186/s13020-025-01171-5

**Published:** 2025-07-11

**Authors:** Shenglan Liu, Junhong Wu, Hao Huang, Bin Hu, Dong Xie, Fuqiang Cao, Jingxuan Li, Caiyao Guo, WeiJie Peng, Yanli Jin, Wei Dai

**Affiliations:** 1 Jiangxi Province Key Laboratory of Pharmacology of Traditional Chinese Medicine, Gannan Medical University, Ganzhou, 341000 China; 2 Jiangxi Provincal Key Laboratory of Tissue Engineering, School of Pharmacy, Gannan Medical University, Ganzhou, 341000 China; 3 School of Rehabilitation Medicine, Gannan Medical University, Ganzhou, 341000 China; 4 Jinan University Institute of Tumor Pharmacology, College of Pharmacy, Jinan University, Guangzhou, 510632 China

**Keywords:** Solamargine, Colorectal cancer, Hepatic metastasis, Ferroptosis, Cancer stem cells

## Abstract

**Background:**

Colorectal cancer (CRC) is a prevalent malignant tumor globally, ranking third in incidence and second in mortality. Metastasis is the main cause of death in patients with CRC. *Solanum nigrum* L. (SNL), a traditional Chinese medicinal herb endowed with detoxification, blood circulation enhancement, and anti-swelling properties, has been widely used in folk prescriptions for cancer treatment in China. Solamargine (SM) is the major steroidal alkaloid glycoside purified from SNL. However, its role and mechanism against metastatic CRC are not yet clear. The purpose of this study was to evaluate the inhibitory effect of SM on human hepatic metastatic CRC and investigate its underlying mechanism.

**Methods:**

CCK-8 assay, colony-formation assay, transwell assay, flow cytometry, tumoursphere formation assay, reverse-transcription quantitative PCR (RT-qPCR), Western blotting, transcriptomic sequencing and ferroptosis analysis were performed to reveal the efficacy and the underlying mechanism of SM in CRC cell lines. In vivo, allograft model, patient-derived xenograft (PDX) model, and liver metastatic model were performed to verify the effect of SM on the growth and metastasis of CRC.

**Results:**

SM was found to suppress hepatic metastasis in CRC by effectively targeting key cellular processes, including proliferation, survival, and stemness. RNA sequencing showed that SM could induce ferroptosis, which was confirmed by elevated lipid reactive oxygen species (ROS) and downregulated glutathione peroxidase 4 (GPX4) and glutathione synthetase (GSS) in CRC cells and xenografts. Induction of ferroptosis by SM was regulated by nuclear factor erythroid 2-related factor 2 (Nrf2). Furthermore, downregulation of β-catenin was found to be fundamental for the SM-enabled cancer stem cells (CSCs) elimination and metastasis blockage in CRC.

**Conclusion:**

Our results indicated that SM is a promising therapeutic drug to inhibit hepatic metastasis in CRC by inducing ferroptosis and impeding CSCs.

**Supplementary Information:**

The online version contains supplementary material available at 10.1186/s13020-025-01171-5.

## Introduction

Colorectal cancer (CRC) is a prevalent malignant tumor globally, ranking the third in incidence and the second in mortality [1]. Metastasis is the main cause of death in patients with CRC. Clinical data reveal that liver metastatic CRC occur in 15% to 25% of patients at initial diagnosis, while 50% to 70% develop liver metastases during the disease progresses [2]. At present, the treatment of primary CRC mainly includes endoscopic therapy, surgical resection and radiotherapy, with emerging integration of targeted agents and immunotherapy. However, once hepatic metastasis occurs, there are no effective treatment methods available. The absence of effective treatment leads to an extremely poor prognosis with a 5-year survival rate of merely 11%, posing a big challenge in the clinical treatment of CRC [3, 4]. Therefore, it is particularly urgent to clarify the mechanism of CRC liver metastasis and explore new therapeutic drugs.

According to the Huangdi Neijing, the major principle of Traditional Chinese Medicine in treating tumors involves detoxification, which aimed at removing pathogenic agents [5]. *Solanum nigrum* L. (SNL) is frequently employed in tumor treatment regimens, renowned for its biological actions such as detoxification, heat dispersion, blood circulation enhancement, and anti-swelling properties [6, 7]. Numerous efforts have been made to extract antitumor agents from SNL, resulting in the isolation of active compounds such as steroidal glycosides glycoproteins [8], glycoalkaloids [9] and polysaccharides [10]. Solamargine (SM), a steroid alkaloid glycoside, is the main active substance isolated from SNL. Recent studies have reported that SM shows excellent antineoplastic efficacy in various malignant tumors (e.g., liver cancer, pancreatic cancer, nasopharyngeal cancer and gastric cancer) [11–13]. However, whether SM possesses activity against metastatic CRC and the underlying mechanisms remain to be explored.

Tumor metastasis is a complex biological process involving multiple steps [14]. Firstly, the primary tumor cells invade surrounding tissues, and then infiltrate into lymphatic or blood vessels to become circulating tumor cells (CTCs). Only a few CTCs acquire stemness to resist death, immune killing, and shearing stress. Upon reaching distant target organs, a small number of viable CTCs exit from blood or lymphatic vessels. These CTCs undergo extravasation and transform into metastasis-initiating cells (MICs) with the ability to survive, colonize, and proliferate in the distant organs, eventually evolving into observable metastases [15]. Studies have shown that both CTCs and MICs have cancer stem cells (CSCs)-like properties [16]. Therefore, cellular characteristics including proliferation, survival, and stemness play a crucial role in facilitating the formation of metastatic lesions in target organs for tumor cells.

A new form of programmed cell death (PCD) termed ferroptosis was first pioneered by Dixon group in 2012 [17]. In contrast to autophagy and apoptosis, ferroptosis is a state of PCD characterized by the accumulation of iron and reactive oxygen species (ROS), primarily attributed to diminished activity of glutathione peroxidase 4 (GPX4), cysteine depletion, and peroxidation of arachidonic acid [18, 19]. Researchers have demonstrated that ferroptosis can significantly enhance the effectiveness of eliminating cancer cells, highlighting its vital role in cancer treatment [20]. Nuclear factor erythroid 2-related factor 2 (Nrf2) contributes to prevent lipid peroxidation and ferroptotic cell death by activating several antioxidant response element-containing genes, including GPX4, an enzyme crucial for converting lipid peroxides into lipid alcohols. Moreover, Nrf2 regulates multiple key enzymes implicated in glutathione synthesis, such as glutathione synthetase (GSS) [21]. Nrf2 has historically been recognized as a tumor suppressor due to its role in mediating cellular defense responses. However, mounting evidence indicates that Nrf2 also functions as an oncogene in the processes of tumor initiation and metastasis [22]. Therefore, the relationship between Nrf2 and tumorigenesis is complicated and may be context-dependent.

In this study, we found that SM potently inhibits liver metastasis of CRC, resulting in a robust suppression of cellular features associated with metastasis, including proliferation, survival, and stemness. Mechanistically, SM induces ferroptosis by downregulating the Nrf2 and downstream targets, GSS and CPX4, leading to inhibition of cellular proliferation and survival. Additionally, downregulation of β-catenin cascade plays a fundamental role in the SM-mediated elimination of CSCs and the blockage of liver metastasis in CRC. These results identify SM as a promising agent against CRC cells and warrant a clinical trial for CRC patients with hepatic metastasis.

## Materials and methods

### Chemicals

**Fig. 1:**
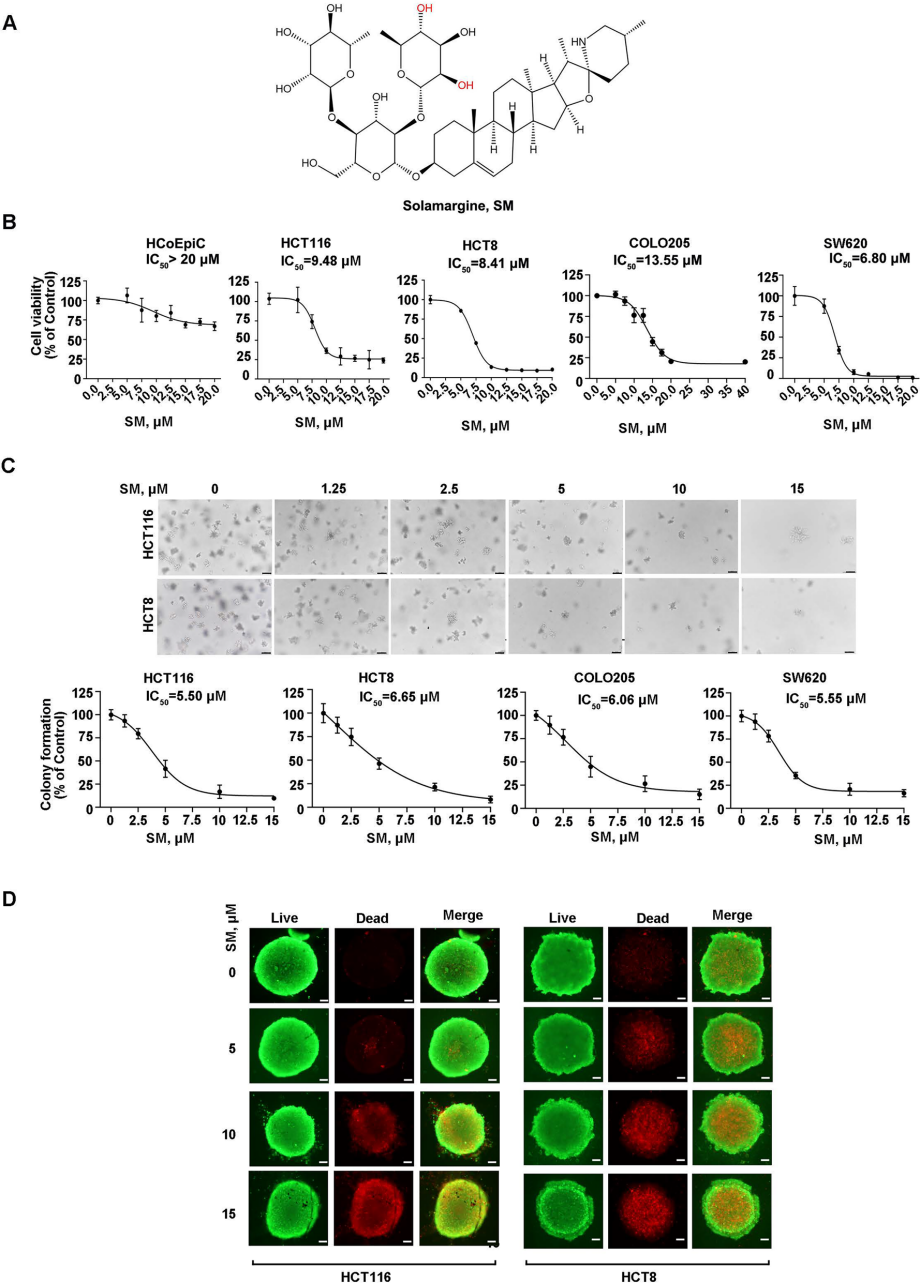
Solamargine (SM) counteracts the activity of colorectal cancer (CRC) cells. **A** Chemical structure of SM. **B** Human CRC cells and normal colonic epithelial cells (HCoEpiC) were treated with ascending concentrations of SM for 68 h, and then cell viability was determined by CCK-8 assay. **C** After pre-treatment with escalating concentrations of SM, CRC cells were cultured in soft agar to evaluate colony-formation ability. Representative images of colonies and quantitative data are shown. Scale bar: 100 μm. **D** The fluorogram of live/dead cells staining of CRC cells following exposure to increasing concentrations of SM. Scale bar: 50 μm

SM (Fig. 1A) [23] purified to ≥ 99.88% was obtained from TargetMol (Shanghai, China), dissolved in DMSO at 20 mM, stored at − 20 °C, and diluted to desired concentrations in the culture medium. Paclitaxel (PTX) and Cycloheximide (CHX) were from Sigma-Aldrich (Shanghai, China). Ferrostain-1, Necrostatin-1, 3-MA, Z-VAD-FMK, and Disulfiram were from TargetMol.

### Cell culture

Normal human colonic epithelial cells, HCoEpiC, were purchased from ScienCell (Carlsbad, CA) and maintained in MEM medium supplemented with 10% fetal bovine serum (FBS, Gibco, NY) and 1% penicillin–streptomycin (Solarbio, Beijing, China). Human CRC cell lines HCT116, HCT8, COLO205 and mouse CRC cell line MC38 were from the American Type Culture Collection (ATCC, Manassas, VA) and maintained with RPMI1640 medium with 10% FBS. Human CRC cell lines SW620 cells and a highly transfectable human embryonic kidney cell line 293 T were obtained from ATCC and cultured in DMEM medium supplemented with 10% FBS. All cells were cultured at 37 °C in a humidified incubator with 5% carbon dioxide. These cells were routinely screened for mycoplasma contamination using a mycoplasma detection kit (FuHeng, Shanghai, China) to confirm their mycoplasma-free status. And cells used in the experiments were all in the logarithmic growth phase. The DMSO concentration in cell culture was maintained less than 0.1% (v/v).

### Cell viability assay

The cell viability was detected according to the protocol of CCK-8 (TargetMol) [24]. In brief, HCoEpiC cells and four CRC cell lines (HCT116, HCT8, COLO205, SW620) were seeded at a density of 5000 cells per well in a 96-well plate overnight and then were exposed to varying concentrations of SM for 68 h. Subsequently, cells were incubated with CCK-8 for 4 h to assess cell viability. Cell viability was calculated using the following formula: Relative viability = (Mean absorbance of treated wells − background absorbance)/(Mean absorbance of untreated wells − background absorbance) × 100%. The half-maximal inhibitory concentration (IC_50_) of SM on normal epithelial cells and four CRC cell lines were determined via curve fitting of the sigmoidal dose–response curve (GraphPad Prism 8, La Jolla, CA).

### Colony-formation assay

The colony-forming ability of CRC cells was determined by using a double-layer agarose (Invitrogen, Guangzhou, China) system as previously described [25]. Briefly, the lower gel was prepared according to the ratio of 2 × RPMI1640 (or DMEM): FBS:1% agarose = 5:1:4, and 400 μL per well was seeded into a 24-well plate. After solidification, 1 × 10^4^ cells/well mixed with 0.5% agarose containing 10% FBS were seeded on a bottom layer of a 24-well plate. Every other day, 200 μL of RPMI1640 or DMEM medium with 10% FBS was added to each well of a 24-well plate. After incubating at 37 °C for a week, clones containing more than 50 cells were photographed and counted using an inverted microscope.

### Three-dimensional spheroids culture of CRC cells

Ten thousand CRC cells in 100 µL of culture medium were seeded into a low-attachment 96-well plate (Corning, NY). After formation of spheroids, the cells were treated with increasing concentrations of SM for 48 h. Subsequently, cell viability was assessed using a fluorescent live/dead assay kit (Solarbio, Beijing, China) according to the previous study [26]. Briefly, cells were incubated with a 1 × assay buffer solution containing propidium iodide (PI) and Calcein-AM at 37 °C in the dark for 30 min. Then images were captured using a fluorescence microscope (LEICA, Germany). Green fluorescence indicates live cells and red fluorescence indicates dead cells.

### Western blotting analysis

CRC cell lines and dissected animal tissues were dissociated with RIPA lysis buffer (0.5% sodium deoxycholate, 1 M NaF, 0.1% SDS, 1 M phosphatase inhibitor, 1% NP-40, 0.1% PMSF and 0.1% protease inhibitor in PBS) to extract total protein, and the BCA protein detection kit (Thermo Fisher Scientific Inc, Waltham, MA) was used to determine those concentrations. After denaturation at 100 °C for 10 min, the samples were separated by SDS-PAGE. Nitrocellulose membranes were used to transfer the separated proteins, blocked with 5% skimmed milk for 0.5 h, and finally incubated with the corresponding antibodies overnight at 4 °C. The following primary antibodies were used for Western blotting: β-catenin (Boster, PA1212), c-Myc (Abmart, T55150), Cyclin D1 (Abcam, ab16663), KLF4 (Proteintech, 11880-1-AP), Nanog (CST, 4903), Oct-4 (CST, 2750), Slug (Proteintech, 12129-1-AP), GSS (Proteintech, 15712-1-AP), GPX4 (Proteintech, 67763-1-Ig), Nrf2 (Proteintech, 16396-1-AP), and β-actin (Sigma-Aldrich, A1978). Membranes were then incubated with the secondary HRP-conjugated goat anti-mouse IgG (Boster, BA1050) and anti-rabbit IgG (Boster, BA1054). Protein bands were visualized by ECL chemiluminescence (MIKX, MK-01000) and analyzed by Image Pro Plus software.

### Transwell assays

The migration and invasion abilities of CRC cells were detected by transwell chamber [27]. For migration assay, 5 × 10^4^ CRC cells resuspended in 200 μL of FBS-free RPMI1640 medium were placed in the upper compartment of the transwell chamber. In the lower compartment, 400 μL of RPMI1640 medium containing 20% FBS was added. After 24 h, the chambers were removed, fixed with 4% paraformaldehyde, stained with 0.5% crystal violet, and cells in the upper chamber were removed. Subsequently, the cells in three randomly selected fields from the lower chamber were captured and quantified using an inverted microscope. For the invasion assay, the experimental steps are basically the same as the migration experiment, the difference is that the upper chamber is pre-coated with 20% Matrigel (Corning, NY).

### PI exclusion assay

After exposure to escalating concentrations of SM, CRC cells were collected and stained with PI, followed by immediate analysis of cell death using flow cytometry.

### Detection of lipid peroxides by flow cytometry

The CRC cells with different treatment were incubated with 1 μM BODIPY™ 581/591 C11, a precise sensor for lipid peroxides that mediate ferroptosis, for 30 min. After incubation, the cells were collected, washed twice with PBS, and then subjected to detection by flow cytometry [28].

### Aldehyde dehydrogenase assay

Aldehyde dehydrogenase (ALDH) assay was carried out according to the instructions of the ALDEFLUOR™ kit (Stem Cell Technologies, BC, Canada) [29]. In brief, 1 × 10^5^ CRC cells were resuspended in ALDEFLUOR™ assay buffer containing ALDEFLUOR™ reagent with or without the specific ALDH enzyme inhibitor DEAB. After incubating in a water bath at 37 °C for 45 min in the dark, cells were washed and resuspended in ALDEFLUOR™ assay buffer, and the proportion of ALDH positive cells was detected by flow cytometry.

### Tumorsphere culture

Following exposure to SM, 5000 alive CRC cells were resuspended in DMEM/F-12 medium (Gibco, NY) supplemented with 1 × B27, 10 ng/mL bFGF, and 20 ng/mL EGF, before being seeded into a 24-well Ultra-low attachment plate (Corning, NY). One week later, tumorspheres (≥ 50 cells) were photographed and counted with an inverted microscope. The cells were collected and replated for three generations for statistical analysis [30].

### Patient-derived xenograft (PDX) model

Under informed consent from each subject or each subject’s guardian following the institutional policies and the Declaration of Helsinki principles, we obtained CRC tissue specimens from fresh surgical specimens at First Affiliated Hospital of Gannan Medical University. The study was approved by the Institutional Research Ethics Committee of First Affiliated Hospital of Gannan Medical University (Approval number: 2023–237). The primary tumors isolated from the patient with CRC were initially implanted into NOD-SCID mice (GemPharmatech, Nanjing, China), and upon reaching a size of ~ 800 mm^3^, the tumors were harvested [31]. These extracted tumors were then fragmented into pieces of 30–50 mm^3^ and re-implanted into NOD-SCID mice for further passages. After three consecutive passages, the resulting tumor xenografts were used for experiments. Tumor fragments of ~ 30 mm^3^ were subsequently implanted into NOD-SCID mice. Once the tumors reached ~ 100 mm^3^, the mice were randomly assigned to receive either vehicle or high dosage of SM (8 mg/kg) for a 14-day period (n = 6).

### Subcutaneous mouse model

Briefly as follows: 3 × 10^6^ MC38 cells in 200 μL PBS were inoculated into the flank subcutaneous tissue of 4–6-week-old male C57BL/6 mice (GemPharmatech, Nanjing, China). When the tumor volume reached ~ 100 mm^3^, the mice were randomly divided into vehicle group and SM group (n = 6). The tumor volume was measured every other day and calculated using the formula 0.4 × a × b^2^ [32], where a is the largest diameter and b is the smallest diameter in millimeters. The mice were intraperitoneally injected with low dosage of SM (4 mg/kg), high dosage of SM (8 mg/kg), positive control paclitaxel (PTX, 5 mg/kg) or vehicle (DMSO: PBS = 1:9) for 5 days on, 2 days off. Two weeks later, the mice were sacrificed by excessive anesthesia and the subcutaneous tumors were removed for weighing, photographing, immunohistochemistry (IHC) and other subsequent experiments.

### Limiting dilution assay

MC38 cells were divided into different density groups (3 × 10^6^, 1 × 10^6^, 3 × 10^5^, 1 × 10^5^ per mouse), and then inoculated into the left flank of C57BL/6 mice. When tumor volumes in control mice with 3 × 10^6^ cells reached ~ 1500 mm^3^, the tumor incidence in each group was measured through L-Calc dilution software (STEM CELL Technologies Inc.) [24].

### Liver metastatic model

The 50 μL PBS containing 5 × 10^5^ MC38-luciferase (MC38-Luc) cells was inoculated into the spleen of C57BL/6 mice [33]. The spleen was removed to reduce the effect of splenic tumor after 10 min. Three weeks later, the bioluminescence signal intensity of the mice and the isolated livers were detected. The livers were fixed with Bouin's solution for one week, the surface nodules were counted, photographed and stained with hematoxylin and eosin (H&E). The animal studies were approved by the Animal Experimental Ethics Committee of Gannan Medical University (Approval number: 2022-181).

### H&E and IHC staining assays

According to standard procedures, samples were fixed with formalin or Bouin's solution and then embedded in paraffin [24]. For IHC assay, the sections were rehydrated for antigen retrieval using the citric acid method, blocked with 3% H_2_O_2_ and 0.5% BSA. Subsequently, the sections were incubated overnight at 4 °C with the primary antibodies against Ki-67 (Proteintech, 27309-1-AP), GSS (Proteintech, 15712-1-AP), GPX4 (Proteintech, 67763-1-Ig), Nrf2 (Proteintech, 16396-1-AP), β-catenin (Boster, PA1212), c-Myc (Abmart, T55150) and Cyclin D1 (Abcam, ab16663), followed by incubation with the secondary antibodies anti-mouse (ZSGB-BIO, PV-9002) and anti-rabbit (ZSGB-BIO, PV-9001). Then, these sections were stained with hematoxylin after reaction with DAB (MXB Biotechnologies, Fuzhou, China). For H&E assay, the rehydrated sections were exposed to eosin for 5–10 min, followed by staining hematoxylin for 1 min. All sections were photographed under an inverted microscope.

### Stable cell lines generation

Scramble (pLKO.1-puro-non-target shRNA), Vector (pCDNA-puro-Vector), pLKO.1-puro-CTNNB1-target shRNA (shβ-catenin) and pCDNA-puro-hCTNNB1 (β-catenin) were purchased from TranSheep Bio (Shanghai, China). pLV3-CMV-NFE2L2 (Nrf2) was from MiaoLing (Guangzhou, China). Lentiviral supernatants were prepared from 293 T cells using a lentiviral packaging system [pCMV-PHR (packaging structure), pCMV-VSVG (enveloping structure)]. Briefly, lentiviral plasmids and packaging constructs were transfected into 293 T cells using polyethyleneimine (Polysciences, Warrington, PA). After 48 or 72 h transfection, the supernatant of lentiviruses was collected, purified with a 0.45 μm sterile membrane filter and used to infect CRC cells with 0.1% polybrene (Sigma-Aldrich, St. Louis, MO). The infected cells were selected with 1 μg/mL puromycin (Sigma-Aldrich) for 7 days [34].

### Reverse-transcription quantitative PCR (RT-qPCR)

Total RNA extraction and RT-qPCR detection were performed as previously described [27]. The mRNA levels of target genes were quantified and reverse-transcribed into cDNA. cDNA was then used for RT-qPCR using SYBR Green PreMix (Tiangen Biotech, Beijing, China) and normalized by GAPDH mRNA levels. The results were analyzed by the 2^−ΔΔCt^ method. Primers were synthesized by Sangon Biotech (Shanghai, China) and listed in Table 1.

**Table 1:** Primers for RT-qPCR

Genes	Forward primers	Reverse primers
*CTNNB1* *GPX4* *GSS* *NFE2L2* *GAPDH*	5′-ATGACTCGAGCTCAGAGGGT-3′ 5′-CTCCATGTCAGGGCCAGTTG-3′ 5′-CCTATGCTGTGCAGATGGACT-3′ 5′-GCAAATGAGGTTTCTTCGGC-3′ 5′-GCAAATTCCATGGCACCGTC-3′	5′-ATTGCACGTGTGGCAAGTTC-3′ 5′-TGCACGGATTAAGAGCCAGG −3′ 5′-CACCAGAGCACTGGGCAAT −3′ 5′-GGTCTTCTGTGGAGAGGATG-3′ 5′-TCGCCCCACTTGATTTTGG-3′

### Chromatin immunoprecipitation (ChIP) assay

The ChIP assay was conducted using the ChIP Assay Kit (Sigma-Aldrich, 3,955,289) [35]. Briefly, 1 × 10^7^ HCT116 cells were cross-linked with 1% formaldehyde and subsequently quenched with 0.125 M glycine for 5 min. Cell sonication was performed to fragment DNA to sizes ranging from 200 to 500 bp. Immunoprecipitation was then conducted overnight at 4 °C utilizing purified anti-Nrf2 antibody (Proteintech, 16396-1-AP) and normal IgG (CST, 2729S). Following this, antibody-bound chromatin complexes were subjected to washing, elution, and reverse cross-linking. To remove RNA and protein from the protein-DNA complexes, RNase A and proteinase K were applied, respectively. Subsequently, DNA purification from each sample was carried out using a DNA purification kit (Beyotime, D0033). The immunoprecipitated DNA was quantified through qPCR analysis, with the specific primers for ChIP-qPCR detailed in Table 2.

**Table 2:** Primers for ChIP-qPCR

Genes	Forward primers	Reverse primers
*GSS* *GPX4*	5′-ATACTGCCCCACATCTTGGC-3′ 5′-CCCCTCAGGTACAAAAGCCA-3′	5′-CACGGTGACTCAGTTCCCAA-3′ 5′-GAGTCCTCCAGTCTCCCGT-3′

### RNA sequencing

Following 12 h incubation of HCT116 cells with 10 μM SM, RNA was isolated using the RNeasy Mini Kit (Qiagen). Sequencing libraries were prepared according to the manufacturer’s instructions with the NEBNext Ultra™ RNA Library Prep Kit for Illumina (NEB). Active transcripts, defined as genes with mean FPKM > 1, were selected for subsequent analysis. Gene expression was visualized through a volcano plot analysis using GraphPad Prism 8. Gene Set Enrichment Analysis (GSEA) was conducted using the standalone desktop program for further analysis [36].

### Statistical analysis

Statistical analysis was performed using GraphPad Prism 8 software. Data were presented as mean ± standard deviation (SD). Comparisons between two groups were performed using two-tailed Student's *t* test. Differences between multiple groups were performed using one-way analysis of variance, post hoc intergroup comparisons by Tukey’s test. Kaplan–Meier survival was analyzed by log-rank test. *p* < 0.05 was considered to be statistically significant.

## Results

### SM suppresses viability of CRC cells

We firstly evaluated the effect of SM on CRC cell viability. The results of CCK-8 assay showed that SM significantly decreased the viability of HCT116, HCT8, COLO205 and SW620 cells, with IC_50_ values of 9.48 μM, 8.41 μM, 13.55 μM, and 6.80 μM, respectively (Fig. 1B). In contrast, SM exhibited less cytotoxicity to human normal colon epithelial cells (HCoEpiC) with IC_50_ value more than 20 μM (Fig. 1B). Moreover, the results of double-layer soft agar assay demonstrated that SM led to an obvious reduction in colony-formation capacity with IC_50_ values were 5.50 μM, 6.65 μM, 6.06 μM, and 5.55 μM for HCT116, HCT8, COLO205, and SW620 cells, respectively (Fig. 1C). In addition, we conducted three-dimensional culture to evaluate the anti-tumor activity of SM. The results revealed that cells cultured in spheroid form maintained high viability, with minimal cell death were observed. However, an increase of dead cells within the spheroid was found as the concentration of SM treatment elevated (Fig. 1D). Collectively, these data indicate that SM effectively restricts the viability and clonogenicity of CRC cells in vitro, while with low cytotoxicity to normal epithelial cells.

### SM diminishes hepatic metastasis of CRC cells

**Fig. 2:**
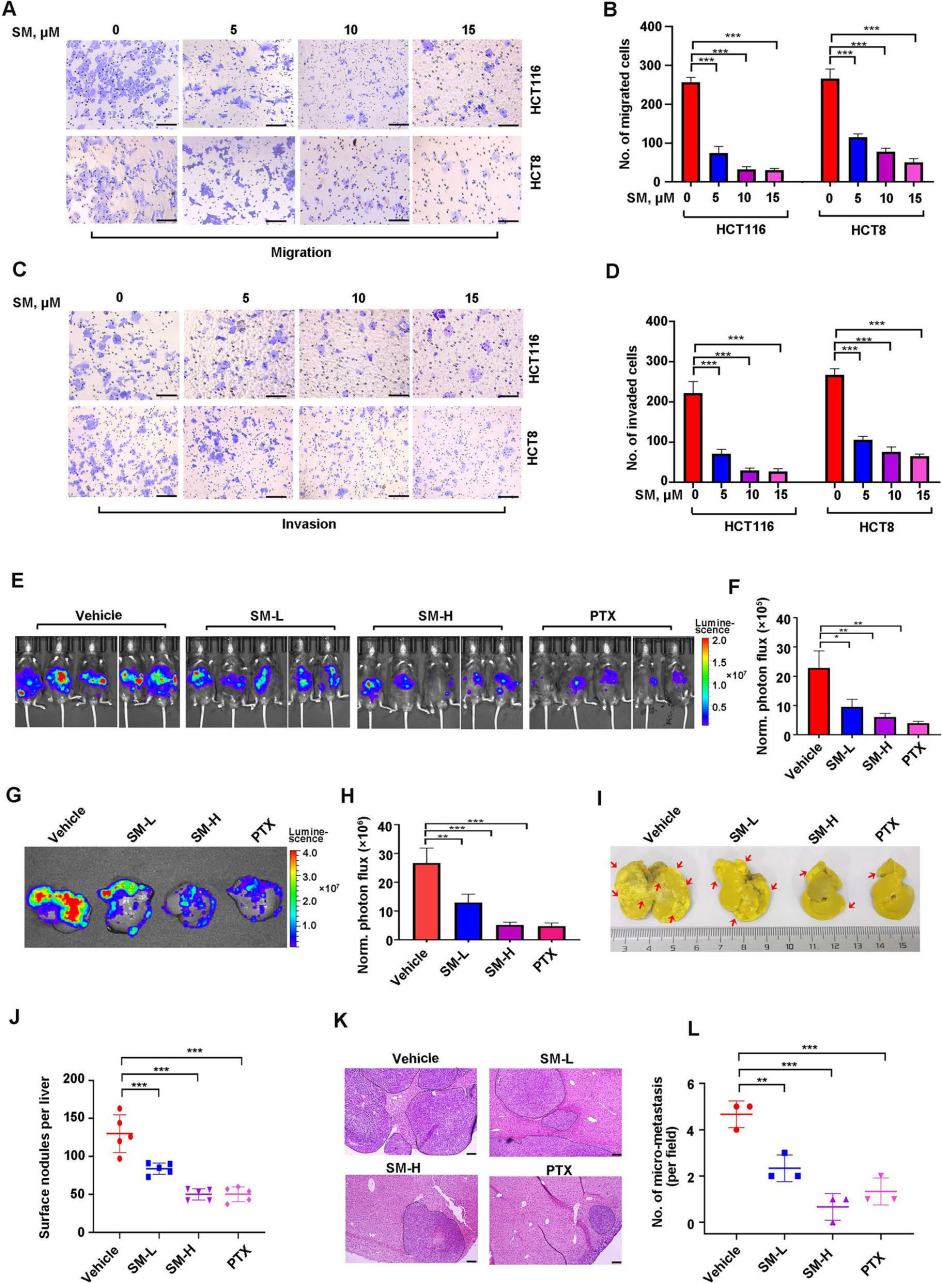
SM attenuates liver metastasis of CRC cells. **A** Representative image of migrated cells and **B** quantitative analysis from 3 random fields after exposure to SM are shown. Scale bar: 100 μm. **C** Representative images of invaded cells and **D** quantitative analysis from 3 random fields after exposure to SM are shown. Scale bar: 100 μm. **E** Photographs of luciferase-based bioluminescent imaging and **F** quantitative analysis of photon flux of MC38-Luc-inoculated C57BL/6 mice following intraperitoneal administration of vehicle (10/90 DMSO in PBS), SM-L (low dosage, 4 mg/kg), SM-H (high dosage, 8 mg/kg), and PTX (paclitaxel, 5 mg/kg) for 3 weeks. n = 5 per group. PTX was used as the positive control. **G** Representative images and **H** quantitative analysis of liver metastasis in C57BL/6 mice (n = 5) measured by bioluminescence imaging. **I** Representative photographs of metastatic livers after Bouin’s solution fixation and **J** quantification of hepatic surface foci in C57BL/6 mice (n = 5). Arrows indicate the presence of metastatic nodules. **K** H&E staining of liver section and **L** quantification of liver metastatic nodules. Scale bar: 200 μm. The values are represented as mean ± SD. *p < 0.05, **p < 0.01, ***p < 0.001

Fifty to seventy percent of patients with CRC develop liver metastases during the course of the disease [37]. Given migration and invasion are essential characteristics for metastasis, the transwell migration and invasion experiments were performed. The results revealed that the migration (Fig. 2A, B) and invasion (Fig. 2C, D) of CRC cells were decreased in response to increasing concentrations of SM treatment. In order to validate the effect of SM on metastasis, an intra-splenic injection liver metastatic model was employed in C57BL/6 mice. The results showed that administration of SM, both in high and low doses, significantly attenuated the bioluminescence signal in the mice (Fig. 2E, F) and in extracted liver samples (Fig. 2G, H), concurrently reducing hepatic surface metastatic nodules (Fig. 2I, J). Consistent with these findings, histological analysis demonstrated a significant reduction in the number of liver metastatic foci in mice treated with both high and low doses of SM compared to those treated with vehicle (Fig. 2K, L). Paclitaxel (PTX), utilized as the positive control, significantly inhibited CRC hepatic metastasis, as evidenced by a decrease in both the bioluminescence signal and the number of liver metastatic foci compared to the vehicle group (Fig. 2E–O). Importantly, no significant weight loss and organ damage (e.g., heart, lung and kidney) were observed in the SM-treated group (Fig. S1A, B). Together, these findings provide strong evidence that SM attenuates liver metastasis in CRC.

### SM attenuates outgrowth of CRC cells in vivo

**Fig. 3:**
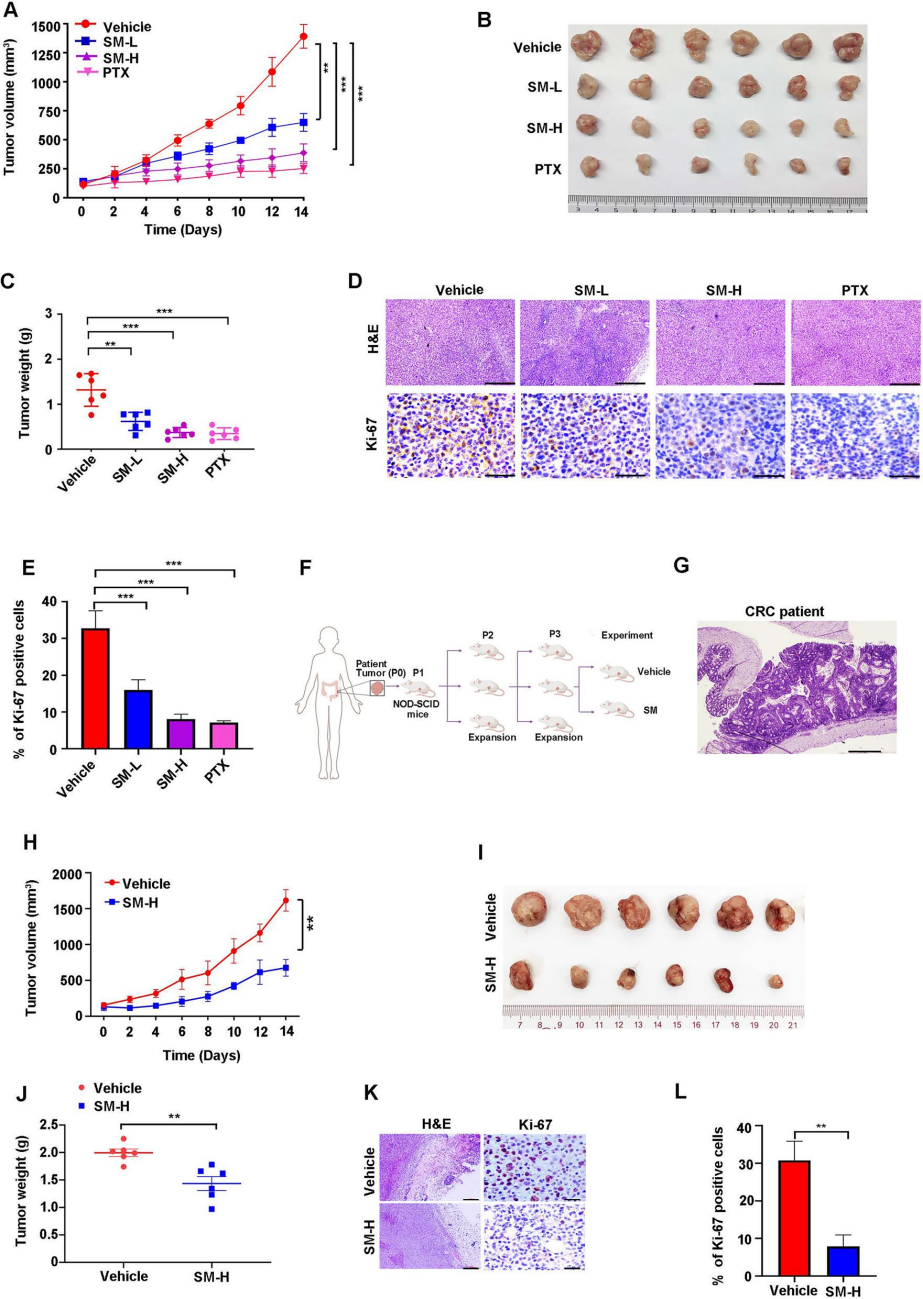
SM restrains the outgrowth of CRC cells in allograft mouse model and patient-derived xenograft (PDX) mouse model. **A** Tumor volume, **B** photographs of excised tumors, and **C** tumor weight of C57BL/6 mice following intraperitoneal administration of vehicle (10/90 DMSO in PBS), SM-L (low dosage, 4 mg/kg), SM-H (high dosage, 8 mg/kg), and PTX (paclitaxel, 5 mg/kg) for 2 weeks, n = 6 per group. **D** Tumor tissues from mice were subjected to H&E staining and immunohistochemical (IHC) analysis using antibody against Ki-67. Scale bar: 200 μm (top), 50 μm (bottom). **E** Quantitative analysis of Ki-67 positive cells in tumor slides from allograft model (n = 3). **F** Flowchart of CRC PDX model. **G** Tumor tissues from CRC patient were subjected to H&E staining, scale bar: 200 μm. **H** Tumor volume, **I** images of excised tumors, and **J** tumor weight of NOD-SCID mice following intraperitoneal administration of vehicle or SM-H for 2 weeks. **K** PDX tissues from mice were subjected to H&E staining and IHC analysis using antibody against Ki-67. Scale bar: 200 μm (left), 50 μm (right). **L** Quantitative analysis of Ki-67 positive cells in tumor slides from PDX model (n = 3). The values are represented as mean ± SD. *p < 0.05, **p < 0.01, ***p < 0.001

Given SM effectively impedes CRC hepatic metastasis, we wonder which metastatic-associated cellular features (e.g., proliferation, survival, CSCs) were dampened by SM. Hence, the effect of SM on proliferation was evaluated in vivo. Remarkably, administration of SM, both in high and low doses, markedly slowed tumor growth, as evidenced by tumor volume (Fig. 3A) and tumor weight (Fig. 3B, C) compared to the vehicle group. Moreover, IHC staining demonstrated a dose-dependent reduction in Ki-67-positive cells following SM treatment (Fig. 3D, E). PTX, served as the positive control, significantly hampered CRC outgrowth, as evidenced by the decrease in tumor volume, tumor weight and proliferation marker Ki-67 compared to the vehicle group (Fig. 3A–E).

PDX model is widely recognized as the most relevant for assessing the in vivo anticancer effectiveness during preclinical drug development [38]. Therefore, CRC PDX model was selected to evaluate the anti-tumor efficacy of SM (Fig. 3F, G). Considering the rarity and limited number of PDX tumors, we only used high-dose of SM for the experiments. The data showed that SM treatment notably reduced tumor volumes (Fig. 3H), tumor weight (Fig. 3I, J) and the proportion of Ki-67 positive cells (Fig. 3K, L), aligning with the findings in the allograft mouse model (Fig. 3A–E). Together, these data suggest that SM hampers the in vivo outgrowth of CRC cells.

### SM induces ferroptotic death of CRC cells

**Fig. 4:**
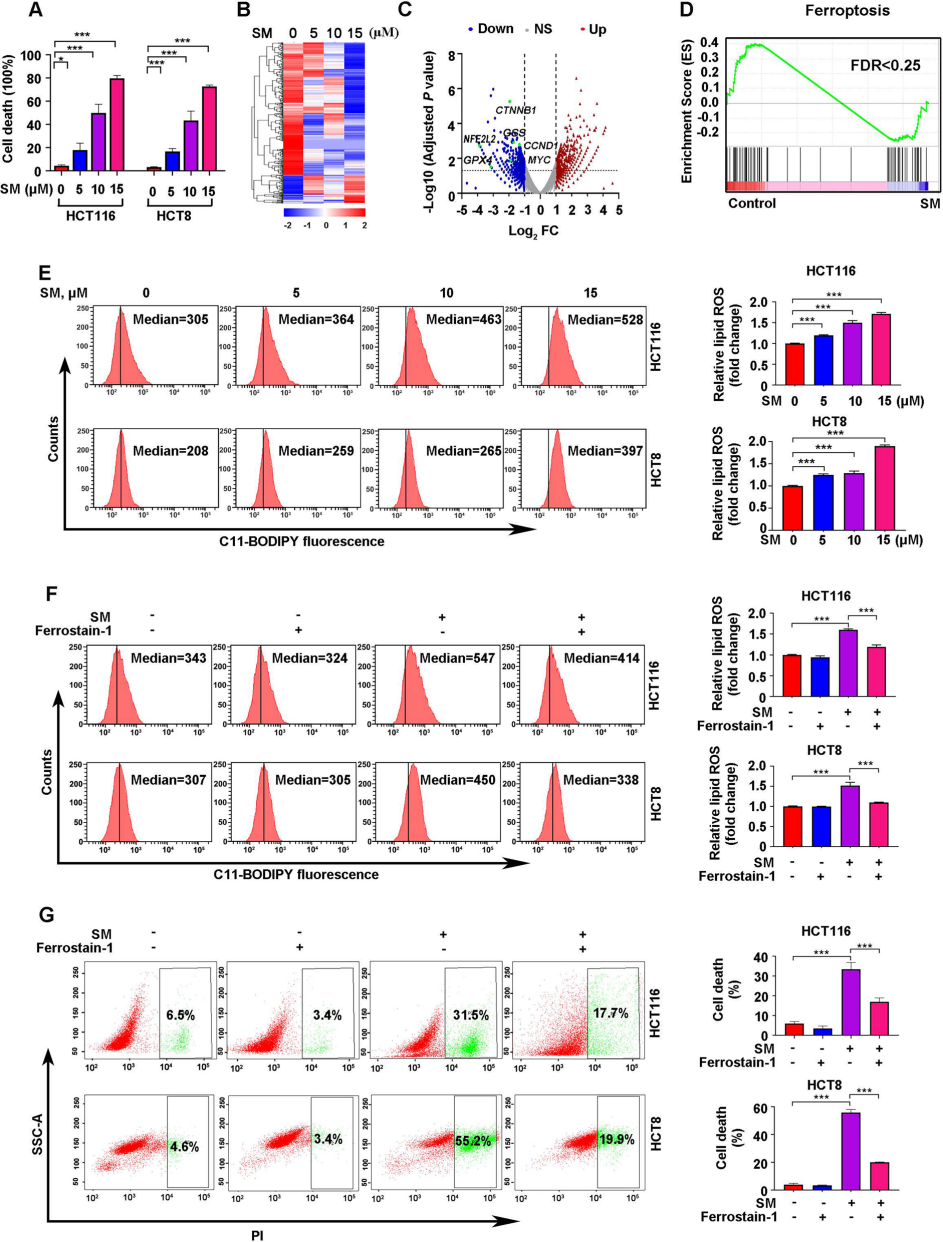
SM promotes ferroptosis in CRC cells. **A** After treatment with SM, CRC cells were stained with PI, and the percentage of cell death was evaluated through flow cytometry analysis. **B** Heatmap displayed the log2 fold expression changes of transcripts in HCT116 cells exposure to increasing concentrations of SM for 12 h. **C** Volcano plots showed the differentially expressed genes in SM-treated HCT116 cells compared to control. **D** GSEA revealed that SM-downregulated genes were significantly enriched in ferroptosis. **E** Representative flow cytometry images and quantitative data of lipid ROS levels are shown following SM treatment of CRC cells. **F** Representative flow cytometry images and quantitative data of lipid ROS levels are shown following SM or ferrostatin-1 treatment of CRC cells. **G** Following treatment with SM or ferrostatin-1, CRC cells were stained with PI, and the percentage of cell death was evaluated by flow cytometry analysis. The values are represented as mean ± SD. *p < 0.05, **p < 0.01, ***p < 0.001

We subsequently investigated whether SM influences cellular survival. The PI exclusion assay demonstrated that SM treatment led to cell death in a dose-dependent way (Fig. 4A). To elucidate the mechanism underlying the SM-medicated cell death in CRC, RNA sequencing was performed. We found that SM elicited dose-dependent alterations in gene transcription (Fig. 4B). Medium concentration of SM (10 μM) was selected for further analysis. A total of 4943 genes showed altered expression levels in SM-treated HCT116 cells compared with control cells. Notably, genes involved in ferroptosis signaling pathway, such as *GPX4*, *GSS* and *NFE2L2*, were significantly downregulated upon SM treatment (Fig. 4B, C). Moreover, GSEA results confirmed that downregulated genes following SM treatment were significantly enriched in the ferroptosis signaling pathway (Fig. 4D).

To validate the results of the RNA sequencing analyses described above, we next explored the impact of SM on ferroptosis, a process marked by the accumulation of lipid ROS [39]. Specifically, we assessed whether SM induces an elevation in lipid ROS levels by employing C11-BODIPY™ staining, a method that specifically detects lipid peroxides [40]. Our results demonstrated a dose-dependent increase in the accumulation of intracellular lipid ROS following SM treatment (Fig. 4E). To further validate that SM could induce ferroptosis, we utilized the ferroptosis inhibitor ferrostatin-1, which removes alkoxyl radicals generated by ferrous iron from lipid hydroperoxides [41]. As expected, the application of ferrostatin-1 successfully reversed the increase of lipid ROS induced by SM treatment (Fig. 4F). Additionally, we assessed cell viability using the PI exclusion assay, and found that ferrostatin-1 partially ameliorated SM-induced cell death (Fig. 4G). Given this partial rescue of ferroptosis inhibition, various death inhibitors were employed to evaluate their ability to mitigate SM-induced cytotoxicity in CRC cells. Results showed that ferroptosis (ferrostatin-1) and apoptosis (Z-VAD-FMK) inhibitors partially rescued SM-mediated cell death, with ferroptosis blockade showing superior efficacy. Notably, combined application of both inhibitors almost completely reversed SM-induced cytotoxicity, whereas inhibitors targeting autophagy (3-MA), necroptosis (Necrostatin-1), or pyroptosis (Disulfiram) failed to confer protection (Fig. S2A-B). These data establish ferroptosis emerges as the principal cell death mechanism executing SM cytotoxicity in CRC cells.

### SM induces ferroptosis in CRC cells via regulating the level of Nrf2 signaling

**Fig. 5:**
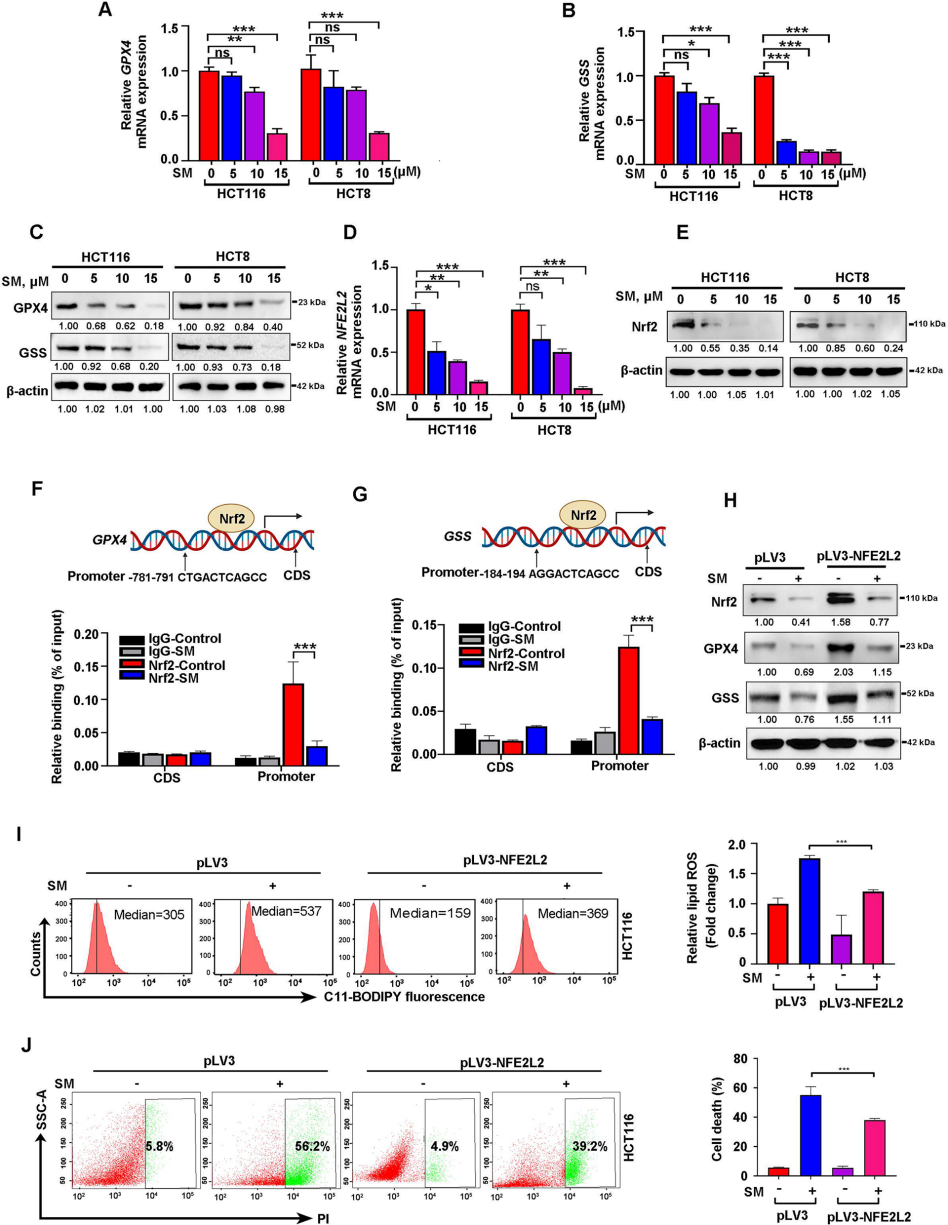
SM induces ferroptosis through suppressing Nrf2 signaling in CRC cells. **A, B** The mRNA levels of *GPX4* and *GSS* in CRC cells were determined by RT-qPCR. **C** The protein levels of GPX4 and GSS in CRC cells were analyzed using Western blotting. **D, E** The mRNA and protein expression levels of Nrf2 (encoded by the *NFE2L2* gene) in CRC cells following SM treatment. **F** ChIP-qPCR analysis revealed the specific binding of Nrf2 to the promoter region rather than the coding sequence (CDS) of *GPX4* in CRC cells. The sequence represents Nrf2-binding site at the promoter of *GPX4* gene predicted by JASPAR (https://jaspar.elixir.no/). Primer for CDS region was served as negative control. **G** ChIP-qPCR analysis revealed the specific binding of Nrf2 to the promoter region rather than the CDS of *GSS* in CRC cells. Primer for CDS region was served as negative control. **H** Overexpression of Nrf2 counteracted the reduced expression of GPX4 and GSS induced by SM in CRC cells. **I** Nrf2 overexpression rescued the accumulation of ROS induced by SM. **J** Overexpression of Nrf2 reversed SM-mediated cell death in CRC cells. The values are represented as mean ± SD. ns, not significant, *p < 0.05, **p < 0.01, ***p < 0.001

Emerging evidence suggests that GPX4 plays an important role in prevent ferroptosis by inhibiting lipid peroxidation [42]. Meanwhile, GSS promotes the production of reduced glutathione, a powerful antioxidant, significantly restrain the ferroptosis process [43]. In light of these functions, we examined the key ferroptosis markers GPX4 and GSS using RT-qPCR and Western blotting. Consistent with previous study [42], our data also revealed decreased expression of GPX4 and GSS at both mRNA and protein levels following SM treatment, corroborating the activation of ferroptosis (Fig. 5A–C).

In parallel, we examined the role of Nrf2, a key antioxidant factor known to promote the transcription of GPX4 and GSS to suppress ferroptosis [44, 45]. Our finding revealed that SM treatment significantly suppressed the mRNA and protein levels of Nrf2 (encoded by *NFE2L2* gene) in CRC cells (Fig. 5D, E). Subsequently, a CHX chase assay was performed to assess the effect of SM on Nrf2 protein turnover. Inhibition of de novo protein synthesis by CHX failed to accelerate Nrf2 degradation in SM-treated CRC cells relative to controls (Fig. S3A, B), which demonstrated that SM downregulates Nrf2 expression primarily through transcriptional suppression rather than enhanced protein degradation. To further directly confirm whether GPX4 and GSS are transcriptionally regulated by Nrf2 in CRC, we conducted ChIP-qPCR assay. The results demonstrated significant recruitment of Nrf2, but not IgG, to the promoters of *GPX4* and *GSS* genes. SM treatment markedly reduced Nrf2 binding at these promoter regions without affecting the coding sequence (CDS) regions, indicating a direct effect on transcription initiation (Fig. 5F, G). In addition, ectopic overexpression of Nrf2 not only restored the suppressed expression of GPX4 and GSS induced by SM (Fig. 5H) but also reversed the consequent lipid ROS accumulation (Fig. 5I) and cell death (Fig. 5J). Moreover, consistent with the results of in vitro cell experiments, IHC analyses demonstrated that SM remarkably downregulated the expression of anti-ferroptosis-related proteins Nrf2, GPX4 and GSS in allograft mouse model and PDX model (Fig. S3C-F).

Further analyses of The Cancer Genome Atlas (TCGA) database showed that high Nrf2 expression was associated with reduced overall survival and shortened disease-free survival in CRC patients (Fig. S4A, B). Similarly, the clinical data from TCGA revealed that the expression of GPX4 and GSS were remarkably upregulated in CRC tissues relative to normal colorectal tissues (Fig. S4C, D). Moreover, the mRNA levels of GPX4 and GSS were not only elevated in different stages of CRC compared with normal tissues (Fig. S4E, F), but also positively associated with lymph node metastatic stages of CRC (Fig. S4G, H). In addition, high expression of GPX4 and GSS was significantly associated with a low overall survival rate in patients with CRC (Fig. S4I, J), potentially indicating oncogenic roles for Nrf2 pathway in CRC. Together, these results demonstrate that SM induces ferroptosis by downregulating the Nrf2 signaling pathway, confirming its critical influence on key anti-ferroptosis regulators in CRC.

### SM diminishes the traits of CSCs in CRC

**Fig. 6:**
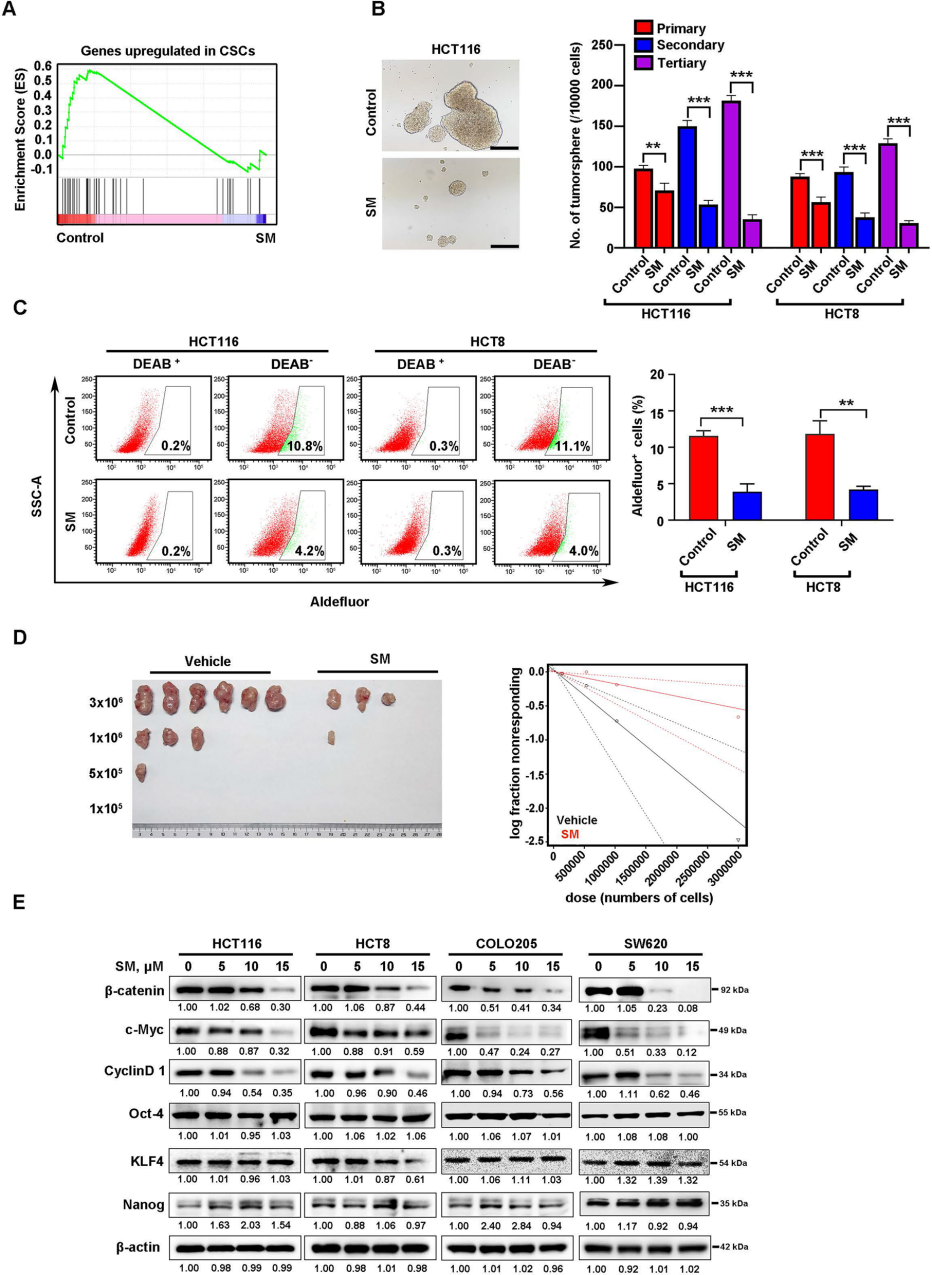
SM treatment despairs the cancer stem-like cells (CSCs) properties in CRC cells. **A** GSEA revealed that genes downregulated by SM were significant enriched in CSCs. **B** SM diminished tumorsphere formation and serially plating capacity of CRC cells. HCT116 and HCT8 cells were collected, resuspended in stem cell medium, and cultured in ultralow-attachment 24-well plates (10,000/well) after treatment with 10 μM SM for 24 h. One week later, the tumorspheres were counted and cells were harvested for the secondary and tertiary rounds of tumorsphere culture, respectively. Representative images of tumorsphere and quantification data are shown. Scale bar: 100 μm. **C** SM decreased the percentage of ALDH^+^ cells in CRC cells. HCT116 and HCT8 cells were incubated with 10 μM SM for 24 h, and ALDH^+^ cells were detected by flow cytometry assay. Representative images of ALDH^+^ cells and quantification data from 3 independent experiments are shown. **D** SM reduced the frequency of CRC CSCs performed by limiting dilution assay in C57BL/6 mice. Representative images of tumors removed from the mice of each group and the frequency of CSCs are shown. n = 6 per group. **E** The expression of stemness-related proteins after SM treatment was examined by Western blotting analysis. The values are represented as mean ± SD. **p < 0.01, ***p < 0.001

Given that transcriptomic data revealed SM-downregulated genes were enriched in stemness (Fig. 6A) and that CSCs are roots of metastasis, we therefore investigated the potential of SM to inhibit CSCs. The effect of SM on the self-renewal of CSCs was examined by conducting tumorsphere formation and serial replication assays. The results indicated that SM significantly reduced both the size and number of tumorspheres, as well as the replating ability of CRC cells (Fig. 6B). Additionally, ALDH is a widely accepted biomarker for CSCs in multiple tumors [46]. Therefore, we employed flow cytometry to determine whether SM decreases the percentage of ALDH^+^ cells. The results revealed that SM diminished the percentage of ALDH^+^ cells in CRC cell lines (Fig. 6C). To further ascertain the effect of SM on CSCs properties, in vivo limiting dilution experiment was performed. The results showed that SM significantly suppressed CSCs frequency approximately 4.1-fold (vehicle: 7.36 × 10^−7^; SM: 1.80 × 10^−7^) in CRC (Fig. 6D and Table 3). These data validate that SM impairs the critical traits of CSCs in CRC.

**Table 3:** Limiting dilution assay in C57BL/6 mice

Engrafted mice		
Cell number 3 × 10^6^ 1 × 10^6^ 5 × 10^5^ 1 × 10^5^ Frequency	Vehicle 6/6 3/6 1/6 0/6 1/1358,022	Solamargine 3/6 1/6 0/6 0/6 1/5545,299

To elucidate the underlying mechanism by which SM effectively impairs the phenotype of CSCs, we examined the protein levels of stemness-associated regulators. We found that SM remarkably downregulated the protein levels of β-catenin and its downstream target proteins c-Myc and Cyclin D1, while other stemness-related proteins (e.g., Oct-4, KLF4 and Nanog) were not significantly affected (Fig. 6E). Consistently, IHC detection of tumor xenografts showed that the levels of β-catenin, c-Myc, and Cyclin D1 were significantly reduced in the mice administered SM compared to those treated with vehicle (Fig. S5A-D). To delve into the molecular mechanism of SM-enable β-catenin downregulation, RT-qPCR assay was conducted. We found that SM dose-dependently decreased the transcript level of β-catenin in CRC cells (Fig. S6A, B). Subsequently, a chase experiment was conducted to investigate whether SM influences the turnover rate of β-catenin protein. The inhibition of de novo protein synthesis with CHX contributed to an increased degradation of β-catenin level in SM-treated CRC cells as compared with control (Fig. S6C, D). Collectively, these data indicate that SM downregulates β-catenin expression through reducing transcription and prompting protein degradation.

### SM-induced β-catenin reduction is crucial for eradicating CSCs in CRC

**Fig. 7:**
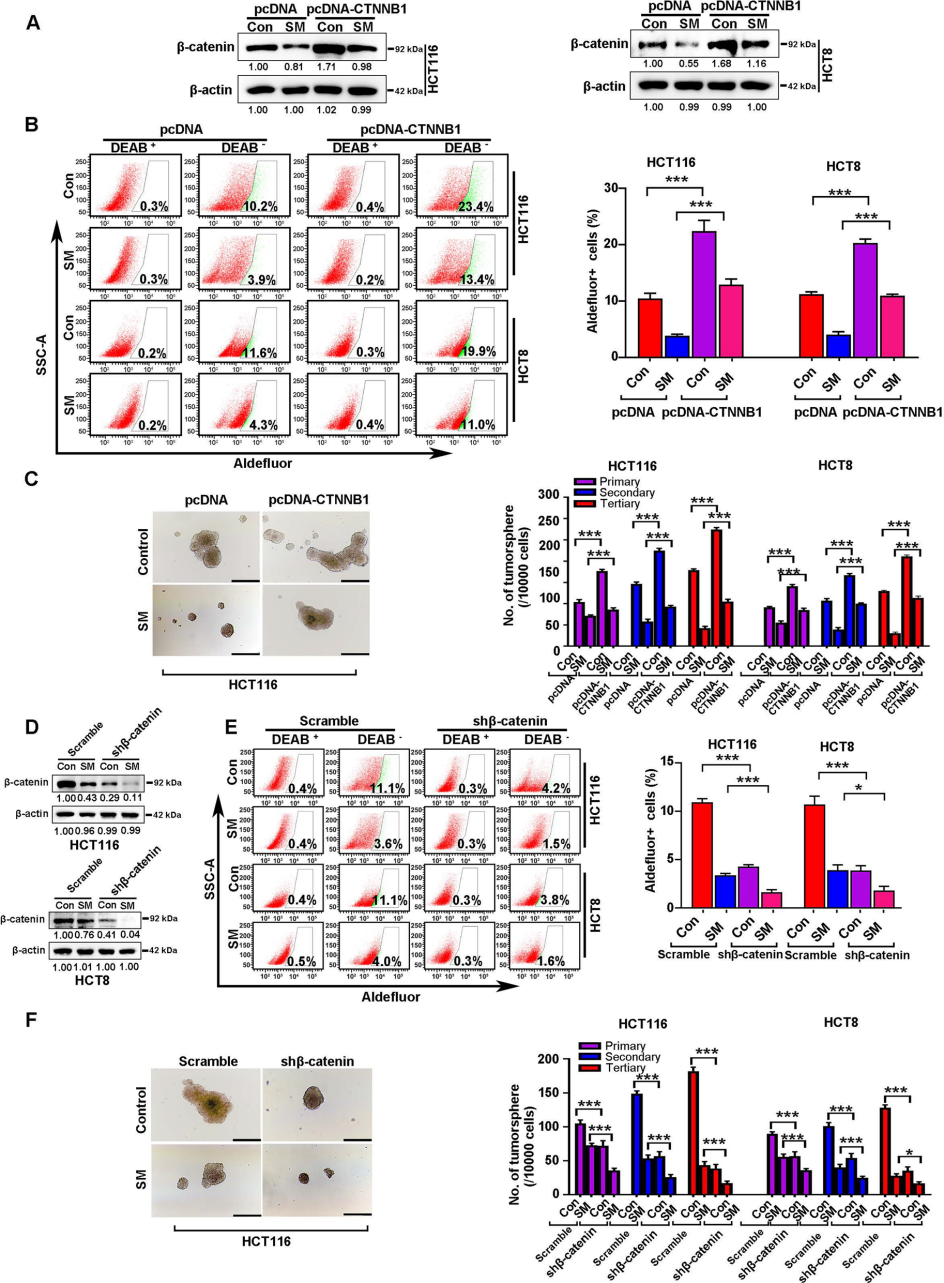
SM impedes traits of CSCs partially through suppressing β-catenin in CRC. **A** Western blotting, **B** ALDH^+^ staining and **C** tumorsphere formation assay were performed after CRC cells transduced with lentiviral β-catenin (encoded by the *CTNNB1* gene) cDNA-expressing construct in the presence or absence of SM. Scale bar: 100 μm. **D** Western blotting, **E** ALDH^+^ staining and **F** tumorsphere formation assays were conducted after CRC cells stably transduced with lentiviral shRNA against β-catenin with or without SM treatment. Scale bar: 100 μm. The values are represented as mean ± SD. *p < 0.05, ***p < 0.001

To further elucidate the role of β-catenin in CRC, HCT116 and HCT8 cells transduced with lentiviral β-catenin-encoding constructs were incubated in presence or absence of SM (Fig. 7A), and then subjected to flow cytometry for ALDH^+^ cells and tumorsphere assay. The results showed that ectopic expression of β-catenin increased the percentage of ALDH^+^ cells (Fig. 7B) and the ability of tumorsphere formation (Fig. 7C). Overexpression of β-catenin attenuated the SM-induced reduction in the percentage of ALDH^+^ cells and the capacity for serial tumorsphere formation (Fig. 7B, C). In contrast, silencing β-catenin by lentiviral shRNA (Fig. 7D) reduced the percentage of ALDH^+^ cells (Fig. 7E) and crippled the capacity of tumorsphere formation in HCT116 and HCT8 cells (Fig. 7F). Depletion β-catenin potentiated the SM-induced reduction in ALDH^+^ cells and serial tumorsphere formation capacity (Fig. 7E, F). Together, these results indicate that the SM-induced downregulation of β-catenin is vital for the elimination of CSCs in CRC.

### β-Catenin counteracts the downregulation of liver metastasis by SM in CRC

**Fig. 8:**
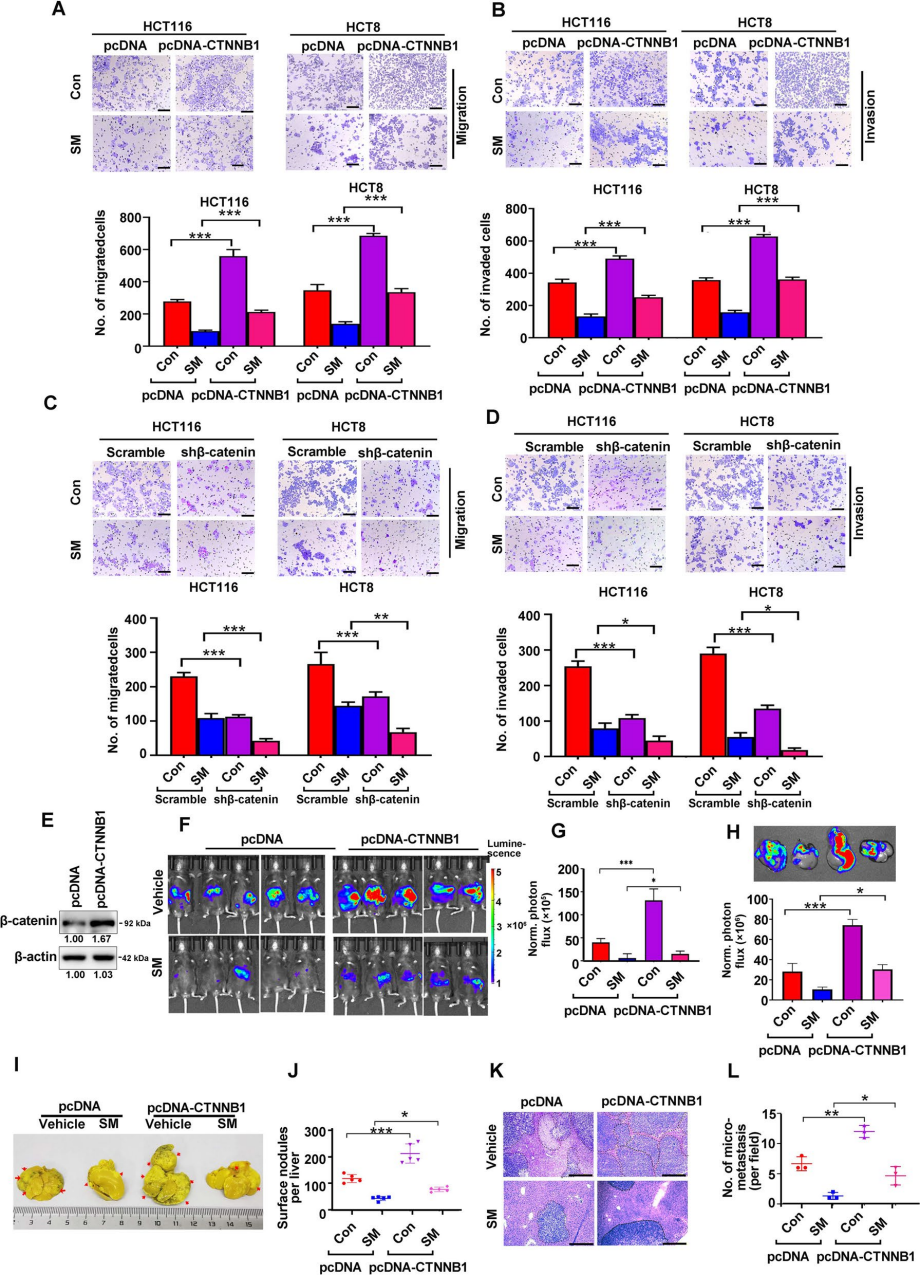
SM inhibits hepatic metastasis of CRC cells partially by attenuating β-catenin. **A** Transwell migration and **B** matrigel invasion assays were conducted followed by CRC cells transduced with lentiviral β-catenin-encoding construct with or without SM treatment. Scale bar: 100 μm. **C** Transwell migration and **D** matrigel invasion assays were conducted followed by CRC cells transduced with lentiviral shRNA against β-catenin in the presence or absence of SM. Scale bar: 100 μm. **E** The overexpression efficiency of β-catenin in MC38-Luc cells was examined by Western blotting analysis. **F** Representative images and **G** quantitative analysis of photon flux on day 21 of C57BL/6 mice followed by intrasplenic injection of MC38-Luc cells stably express β-catenin. n = 5 per group. **H** Representative images and quantitative analysis of liver metastasis in C57BL/6 mice (n = 5) measured by bioluminescence imaging. **I** Representative images of metastatic livers and (**J**) the number of surface foci in the livers in C57BL/6 mice (n = 5) are shown. Arrows indicate the presence of metastatic nodules. **K** Representative photographs of H&E staining of the liver section and **L** the number of liver metastatic foci in microscopic fields are presented (n = 3). Scale bar: 200 μm. The values are represented as mean ± SD. *p < 0.05, ***p < 0.001

Given that β-catenin could promote invasive properties of cancer cells [47], we next investigated the role of downregulated β-catenin in SM-induced blockade of migration and invasion of CRC cells. We found that forced overexpression of β-catenin upregulated the capabilities of migration and invasion (Fig. 8A, B), while depletion of β-catenin decreased the migration and invasion of CRC cells (Fig. 8C, D). Furthermore, the SM-attenuated migration and invasion was significantly reversed by forced expression of β-catenin (Fig. 8A, B), but potentiated by knockdown of β-catenin by shRNA (Fig. 8C, D).

To confirm the role of β-catenin in CRC, an in vivo model of hepatic metastasis was used. We found that overexpression of β-catenin resulted in a notable increase of bioluminescence imaging intensity in mice (Fig. 8E–G) and isolated liver (Fig. 8H), as well as number of metastatic tumor nodules on the surface of livers (Fig. 8I, J). Furthermore, β-catenin overexpression was found to counteract the inhibitory effects of the SM on bioluminescence imaging intensities in mice and liver, as well as on the number of metastatic foci (Fig. 8E–J). Consistently, H&E staining analysis also showed ectopic expression of β-catenin significantly reversed the SM-induced reductions in both size and number of metastatic liver foci (Fig. 8K-L). These results indicate that the downregulation of β-catenin plays a crucial role in the SM-mediated suppression of the CSCs phenotype and hepatic metastasis in CRC cells.

## Discussion

Liver metastasis is recognized as a primary contributor to mortality among patients with CRC [48]. Thus, there is an urgent need to develop novel anti-tumor drugs targeting liver metastasis and to deepen our understanding of the mechanisms underlying their effectiveness. In this study, our findings demonstrated that SM potently inhibits liver metastasis by suppressing key cellular features associated with metastasis in CRC. Specifically, SM reduces cellular proliferation, survival, and stemness, which are critical drivers of tumor progression and metastasis. Furthermore, we identify Nrf2 as an essential mediator for the induction of ferroptosis by SM, contributing to its inhibitory effects on proliferation and survival. Additionally, our research provides evidence that the downregulation of ꞵ-catenin supports the elimination of CSCs by SM and restricts CRC metastasis.

The sustained proliferation and resistance to apoptosis are hallmark characteristics of cancer that significantly contribute to the metastatic process [35]. Our study revealed that SM markedly suppressed the viability of CRC cells in both two-dimensional cultures and three-dimensional cultures. Furthermore, SM inhibited colony-formation, induced cell death, and restrained tumor outgrowth in allograft mouse model and PDX model. These findings are similar with the results in other types of cancer, such as liver cancer [6], lung adenocarcinoma [49] and pancreatic cancer [12].

Mechanistically, we demonstrated that SM induced ferroptosis in CRC cells by downregulating Nrf2 and subsequently decreasing the expression of its downstream genes *GSS* and *GPX4*. Both enzymes are critical in mitigating oxidative stress and, when inhibited, precipitate ferroptosis due to the accumulation of lethal lipid peroxides. Interestingly, solasonine, another active compound isolated from SNL, has shown similar mechanistic effects in hepatocellular carcinoma cells, promoting ferroptosis through downregulating GPX4 and GSS [42]. The parallel findings with solasonine not only substantiate the role of Nrf2 downregulation in mediating ferroptosis but also underscore the therapeutic potential of natural compounds in cancer treatment through this pathway. Mechanistic studies indicate that SM significantly suppresses the transcriptional level of Nrf2, with almost no effect on the protein degradation pathway. Further investigation is needed into the mechanism by which SM inhibits Nrf2 transcription in future.

CSCs represent a small subset of tumor cells characterized by high carcinogenic potential, robust self-renewal, and differentiation capabilities. These cells are widely recognized for their critical role in promoting metastasis [50]. This study firstly demonstrated that SM inhibited the traits of CRC CSCs both in vitro and in vivo, marking a substantial advancement in cancer therapeutics. Furthermore, we found that β-catenin, a critical stemness regulator, can increase the self-renewal potential in CSCs, enhance migration and invasion capabilities, ultimately promote liver metastasis in CRC. Conversely, knockdown of β-catenin led to a significant reduction in these CSC properties and curtailed both cell migration and invasion. Rescue experiments conducted both in vitro and in vivo confirm the critical role of β-catenin as a molecular target for SM mitigating CRC metastasis. Further mechanistic studies reveal that SM not only significantly reduces the transcription levels of β-catenin but also promotes its protein degradation. These discoveries expand our understanding of how SM effectively targets and eliminates CSCs, providing compelling data for its potential application in clinical settings to combat metastatic CRC.

Of importance, previous investigations have demonstrated the selective cytotoxicity of SM. Specifically, SM exhibits more potent growth inhibitory effects against metastatic (WM239A) and primary (WM115) human melanoma cell lines in the vertical growth phase than against the radial growth phase benign melanoma cell line WM35, as well as against normal cell types, including primary bovine aortic endothelial cells, rat fibroblasts, and epithelial cell lines [51]. Consistent with these findings, our study revealed that SM exhibited significant cytotoxic effects on CRC cell lines, while displayed minimal effect on normal colonic epithelial cells. Importantly, in addition to this in vitro efficacy, in vivo studies further validated the suitability of SM for clinical applications. We observed that SM administration did not adversely affect the body weight of mice, and comprehensive histological analyses of kidney, lung, and heart tissues showed no signs of toxicity. These findings not only corroborate earlier studies but also expand the understanding of the differential impacts of SM across cell types [13, 51, 52], reinforcing its potential as a safe and effective therapeutic agent for the treatment of CRC.

Collectively, our findings reveal that SM may decrease the expression levels of Nrf2 and β-catenin, inhibiting the relevant signaling pathways, activating ferroptosis, and diminishing CSCs, ultimately leading to therapeutic effects against CRC. This study validates that SM may serve as a promising therapeutic agent for CRC prevention and treatment. However, the precise molecular mechanisms by which SM downregulates Nrf2 and β-catenin expression require further investigation. Additional experiments on the role of SM in metastasis and its potential targets may provide new insights into effective therapies for CRC.

## Conclusion

SM exhibits robust inhibition of liver metastasis in CRC by effectively targeting key cellular processes, including proliferation, survival, and CSCs, as evidenced by both in vitro and in vivo experiments. Mechanistically, SM induces ferroptosis primarily by suppressing the Nrf2 signaling pathway, resulting in the inhibition of cellular proliferation and survival. Simultaneously, SM suppresses β-catenin at the transcriptional level and facilitates its protein degradation, thus mitigating stemness traits and restraining metastatic progression in CRC. Given the potent antineoplastic properties and multi-targeted mechanisms of SM, the clinical trial of SM in CRC patients with hepatic metastasis is warranted.

## Supplementary Information

Additional file 1

## Data Availability

The data that support the findings of this study are available from the corresponding author upon reasonable request.

## Abbreviations

ALDH: Aldehyde dehydrogenase
ATCC: American type culture collection
ChIP: Chromatin immunoprecipitation
CHX: Cycloheximide
CRC: Colorectal cancer
CSCs: Cancer stem cells
CST: Cell signaling technology
CTCs: Circulating tumor cells
FBS: Fetal bovine serum
GPX4: Glutathione peroxidase 4
GSEA: Gene set enrichment analysis
GSS: Glutathione synthetase
H&E: Hematoxylin and eosin
HCoEpiC: Human normal colon epithelial cells
IC50: Half-maximal inhibitory concentration
IHC: Immunohistochemistry
MICs: Metastasis initiating cells
Nrf2: Nuclear factor erythroid 2-related factor 2
PCD: Programmed cell death
ROS: Reactive oxygen species
RT-qPCR: Reverse transcription quantitative PCR
SM: Solamargine
SNL: *Solanum nigrum* L.
TCGA: The Cancer Genome Atlas
