# Supplementary material for: Effects of solamargine in hepatic metastasis of colorectal cancer: induction of ferroptosis and elimination of cancer stem cells
Source: Chin Med. 2025 Jul 11;20:110. doi: 10.1186/s13020-025-01171-5 (PMC12247209; doi:10.1186/s13020-025-01171-5)

**Supplementary Data**
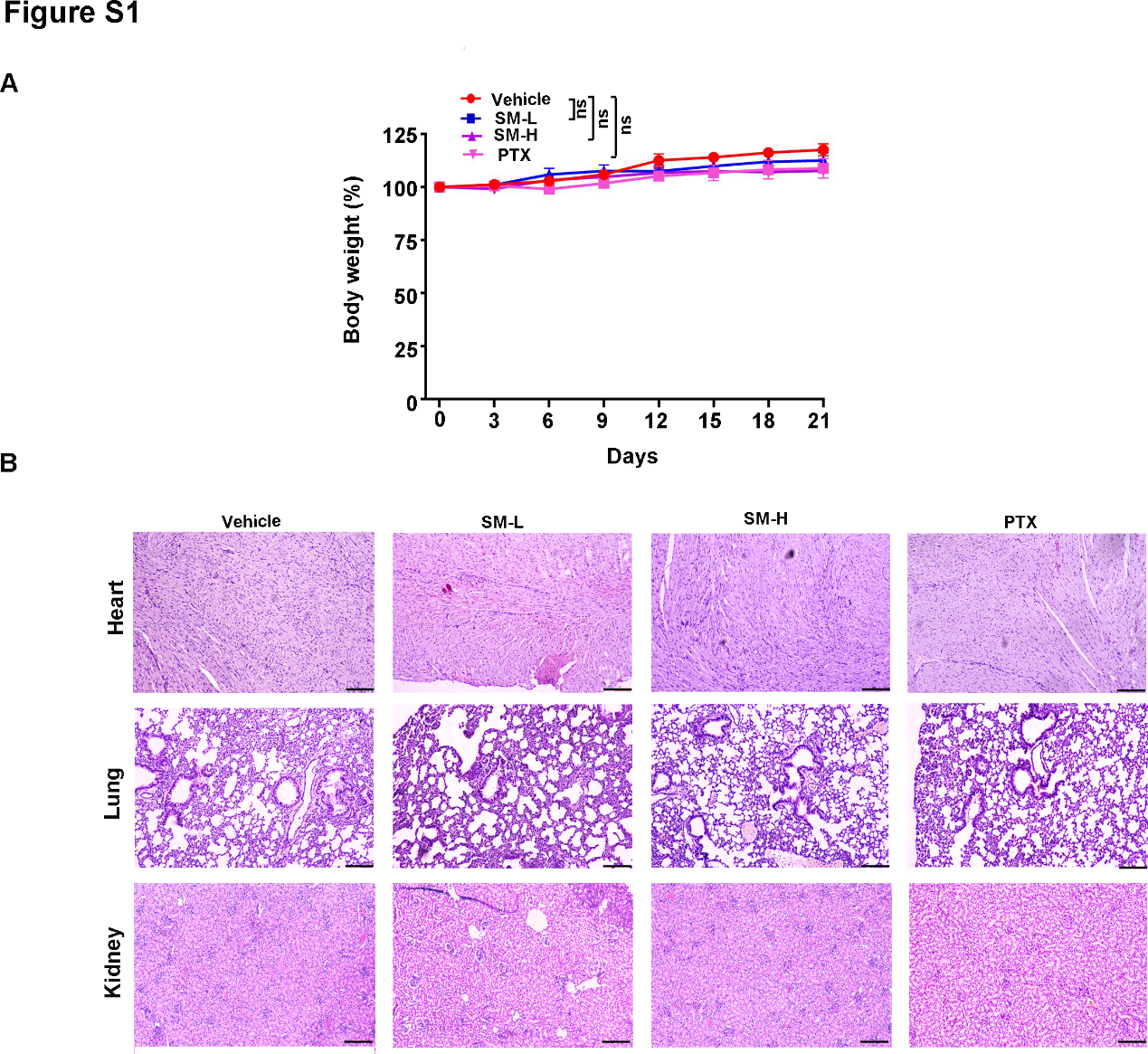


**Fig. S1.** SM exhibits extremely low toxicity to mice. **A** The body weight of each group of mice was recorded every two days, and quantitative analysis of the data was conducted. **B** The tissues from the mice in each group were subjected to H&E staining. The values are represented as mean ± SD, ns means no significance.


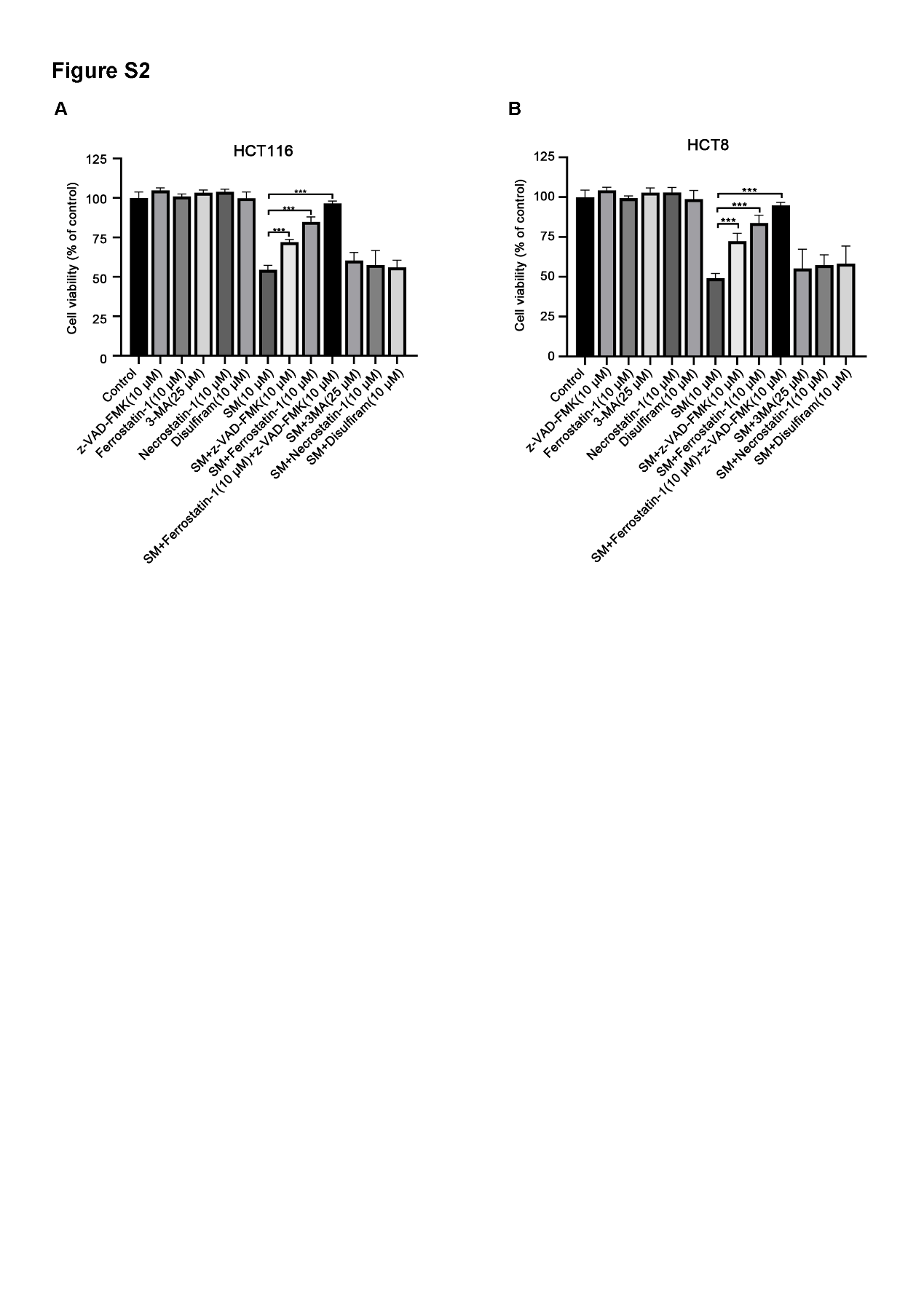


**Fig. S2.** Effect of SM and various cell death inhibitors individually or in combination on viability of HCT116 (**A**) and HCT8 (**B**) cells. The values are represented as mean ± SD. ****P <* 0.001.


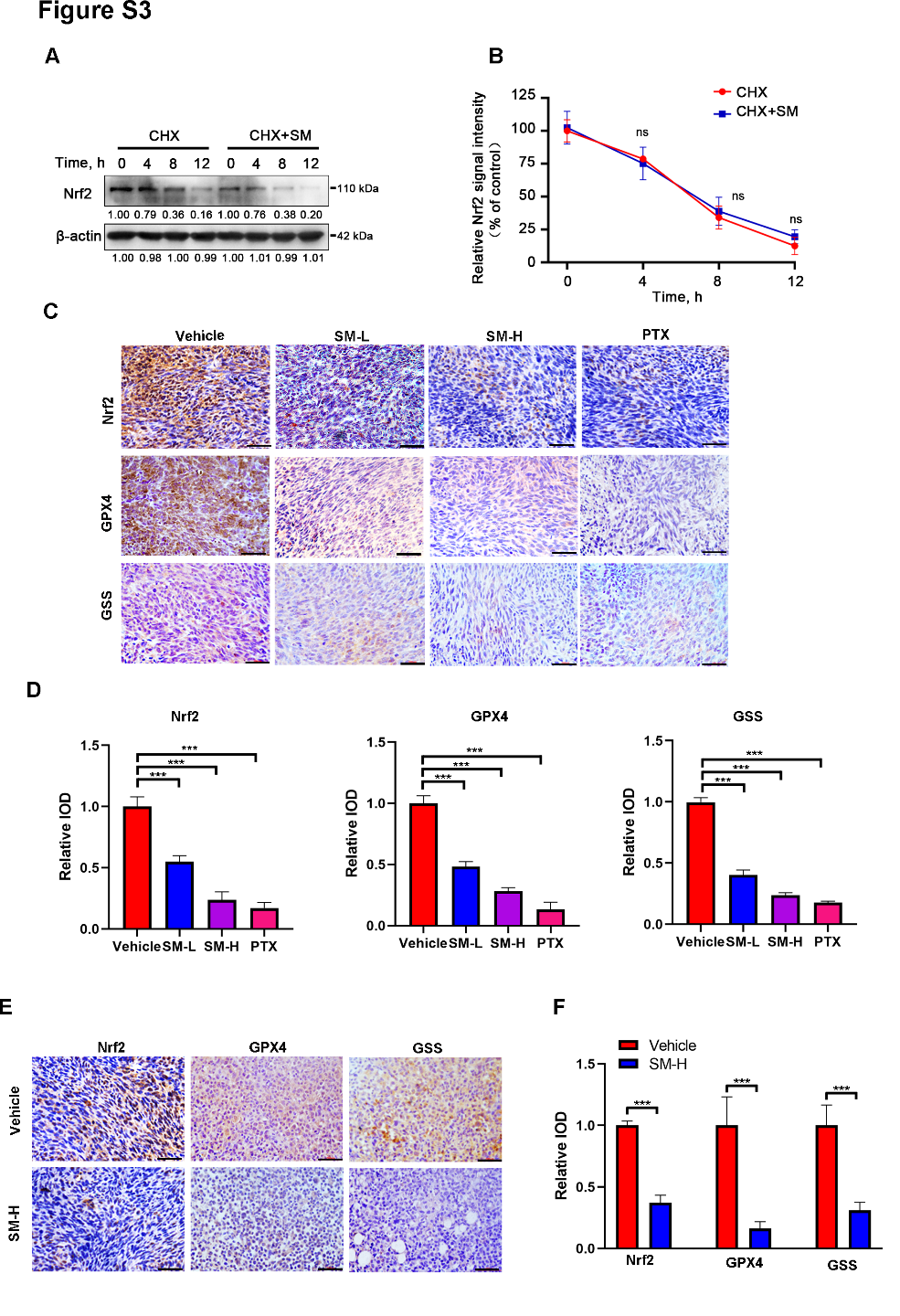


**Fig. S3.** Effect of SM on Nrf2 signaling pathway. **A, B** HCT116 cells were incubated with SM (10 μM) for 12 h and then exposed to cycloheximide (CHX) for various hours, followed by Western blotting assay and densitometric analysis of Nrf2. **C, D** IHC detection of protein levels of Nrf2 signaling axis in MC38 allograft tumors treated with SM or PTX. n=3. Scale bar, 200 μm. **E, F** IHC detection of protein levels of Nrf2 signaling axis in PDX of mice treated with SM-H. n=3. Scale bar, 200 μm. The values are represented as mean ± SD. ns means no significance, ****P <* 0.001.


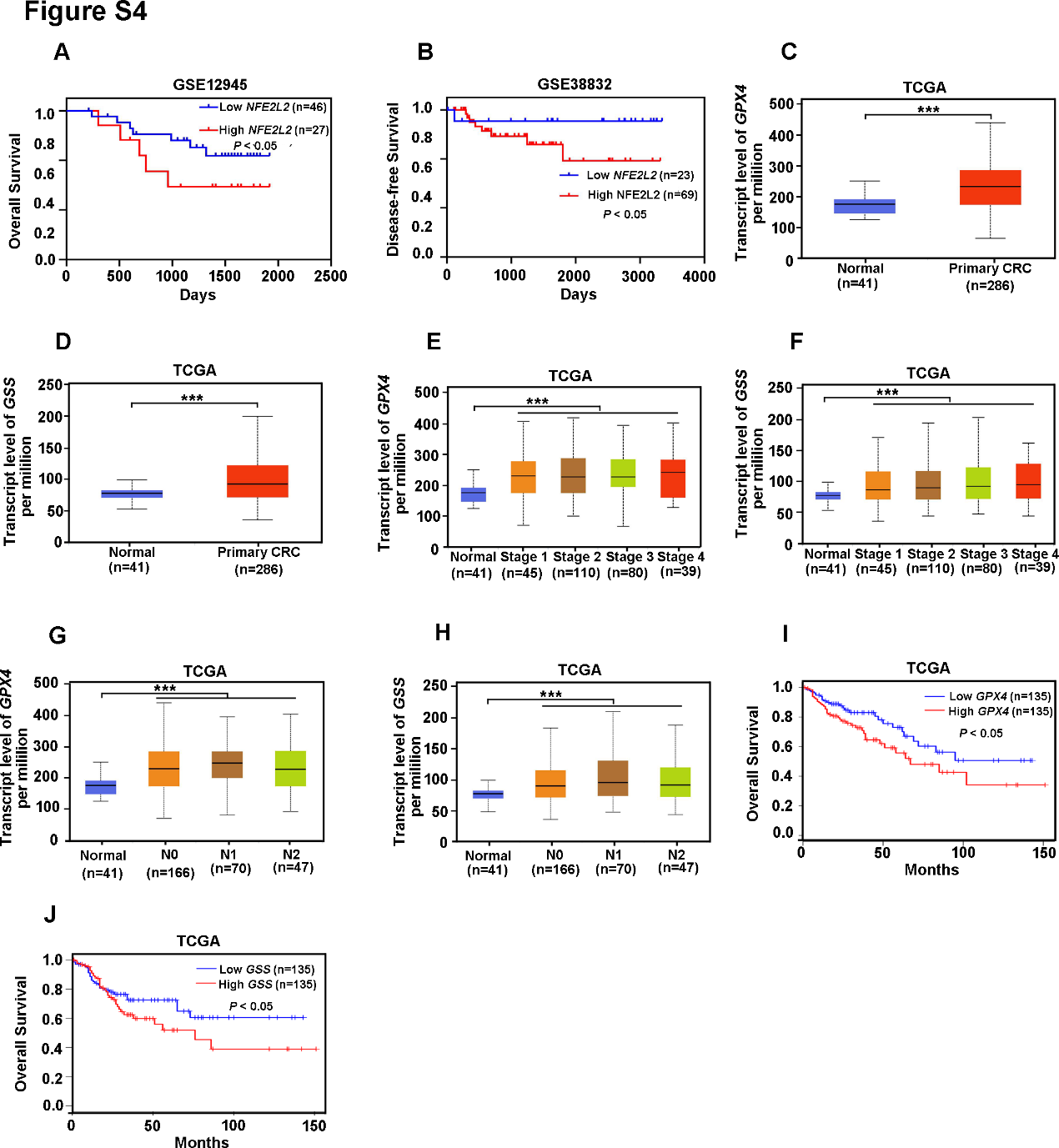


**Fig. S4.** The potential oncogenic roles of Nrf2 signaling pathway in TCGA database. **A, B** Relationship of *NFE2L2* expression with overall survival and disease-free survival in patients with CRC from the cohort of GEO database. **C, D** The mRNA levels of *GPX4* and *GSS* in CRC tissues and normal adjacent tissues were obtained from TCGA database using UALCAN tool (<https://ualcan.path.uab.edu/>). **E, F** The mRNA levels of *GPX4* and *GSS* in different stages of CRC and in normal tissues were derived from TCGA dataset with UALCAN website. **G, H** The transcription levels of *GPX4* and *GSS* were grouped based on the stages of lymph node metastasis in CRC from TCGA database using UALCAN tool. **I, J** The relationship of *GPX4* and *GSS* expression with overall survival in patients with CRC were from TCGA database using GEPIA tool (<http://gepia.cancer-pku.cn/>). The values are represented as mean ± SD. ****P <* 0.001.


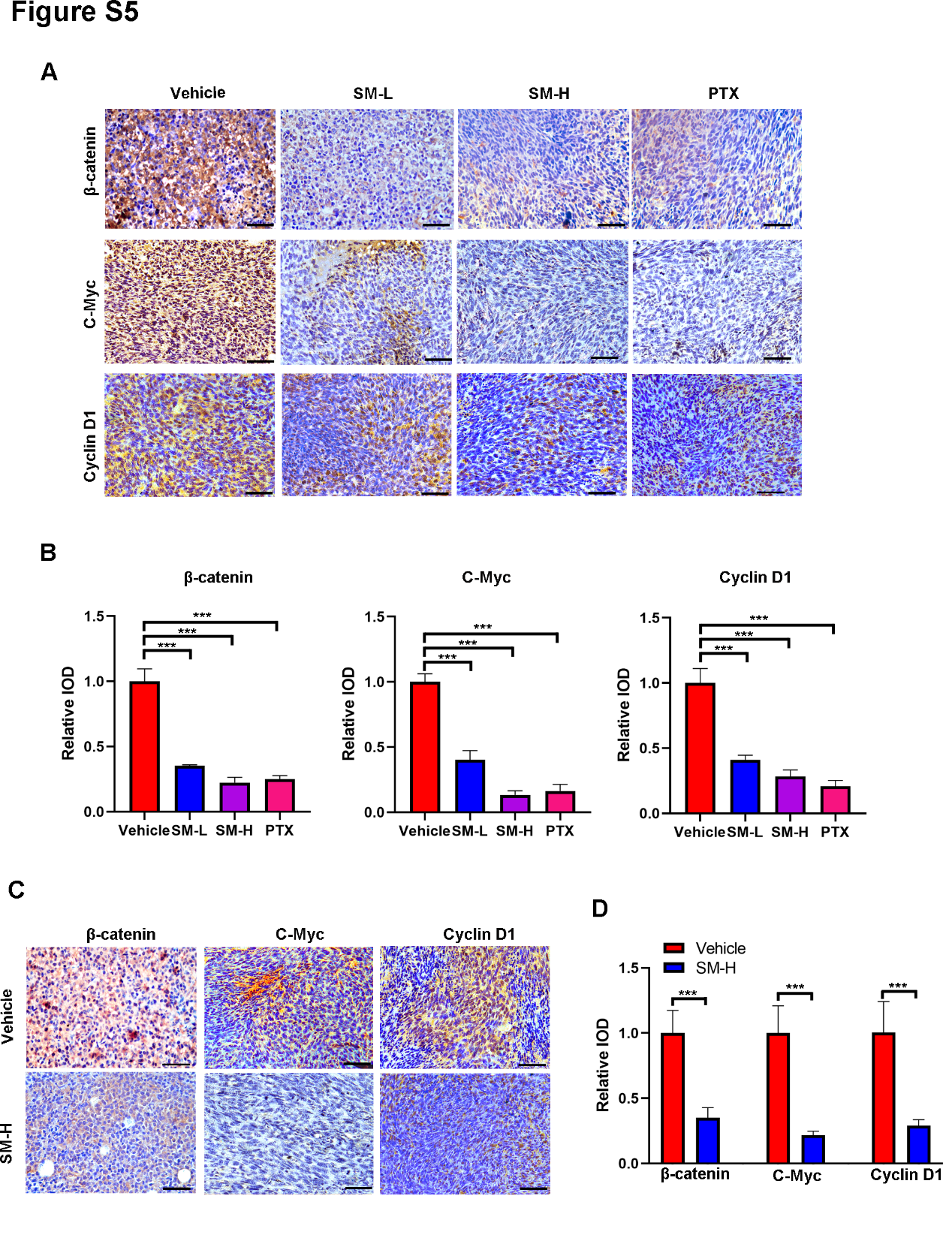


**Fig. S5.** Effect of SM on Wnt/β-catenin signaling pathway. **A, B** IHC detection of protein levels of Wnt/β-catenin signaling axis in MC38 allograft tumors treated with SM or PTX. n=3. Scale bar, 200 μm. **C, D** IHC detection of protein levels of Wnt/β-catenin signaling axis in PDX of mice treated with SM-H. Scale bar, 200 μm, n=3. The values are represented as mean ± SD. ****P <* 0.001.


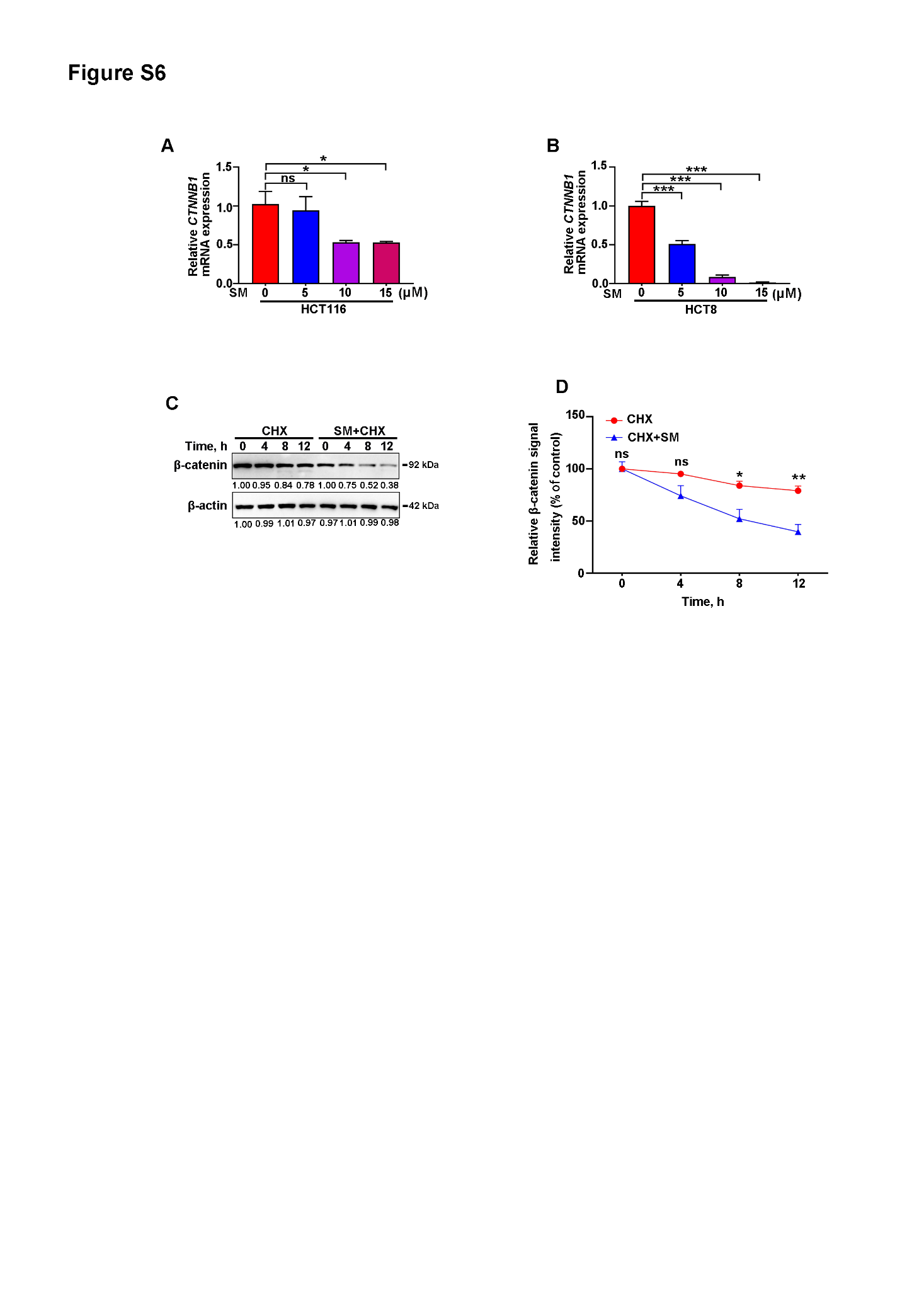


**Fig. S6.** SM downregulates the transcriptional activity and protein stability of β-catenin. **A, B** HCT116 and HCT8 cells were treated with SM, followed by RT-qPCR analysis. **C, D** HCT116 cells were incubated with SM (10 μM) for 12 h and then exposed to CHX for various hours, followed by Western blotting assay and densitometric analysis of β-catenin. The values are represented as mean ± SD. ns, not significant, **P <* 0.05, ***P <* 0.01, ****P <* 0.001.

**Original Western blots**


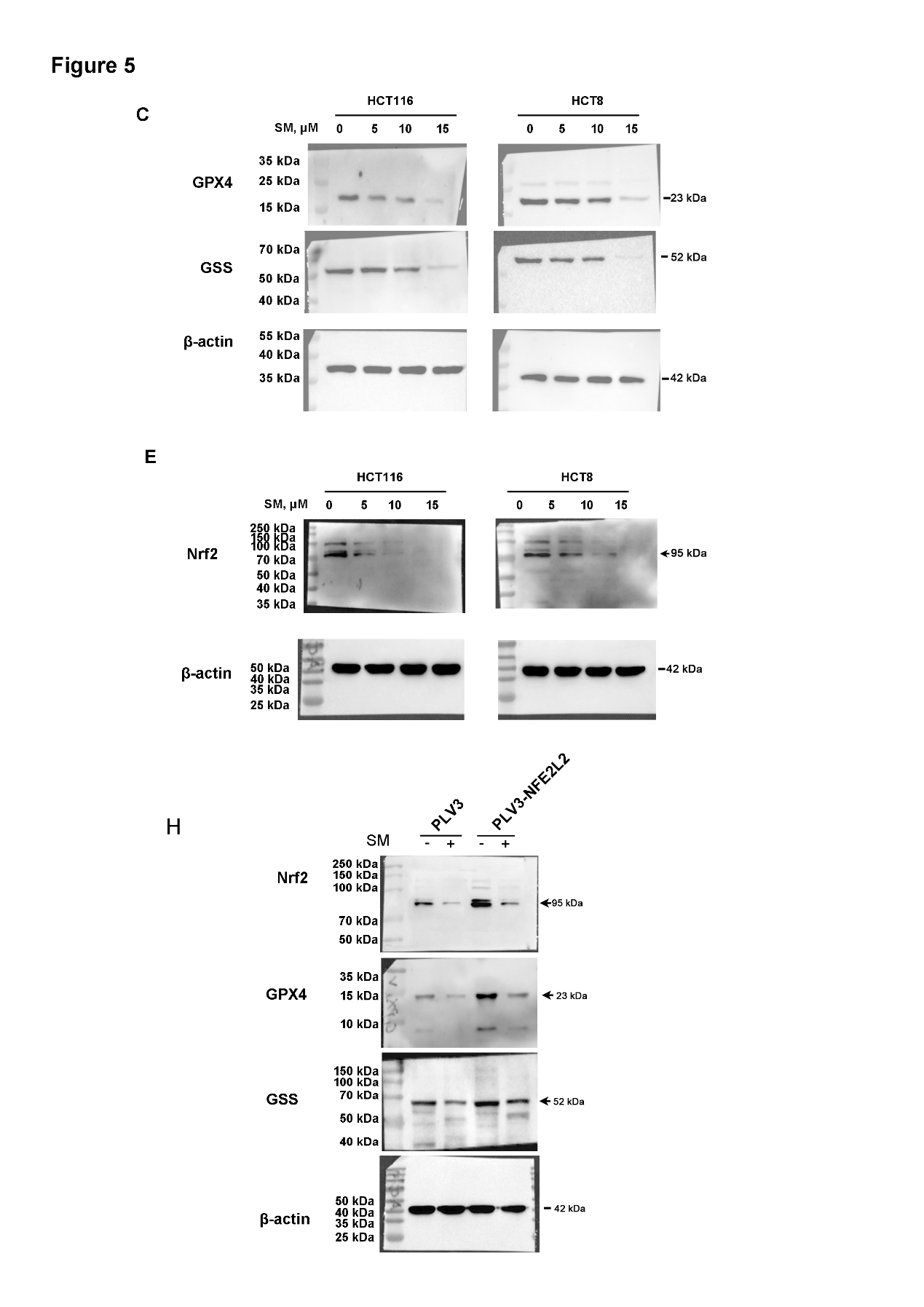


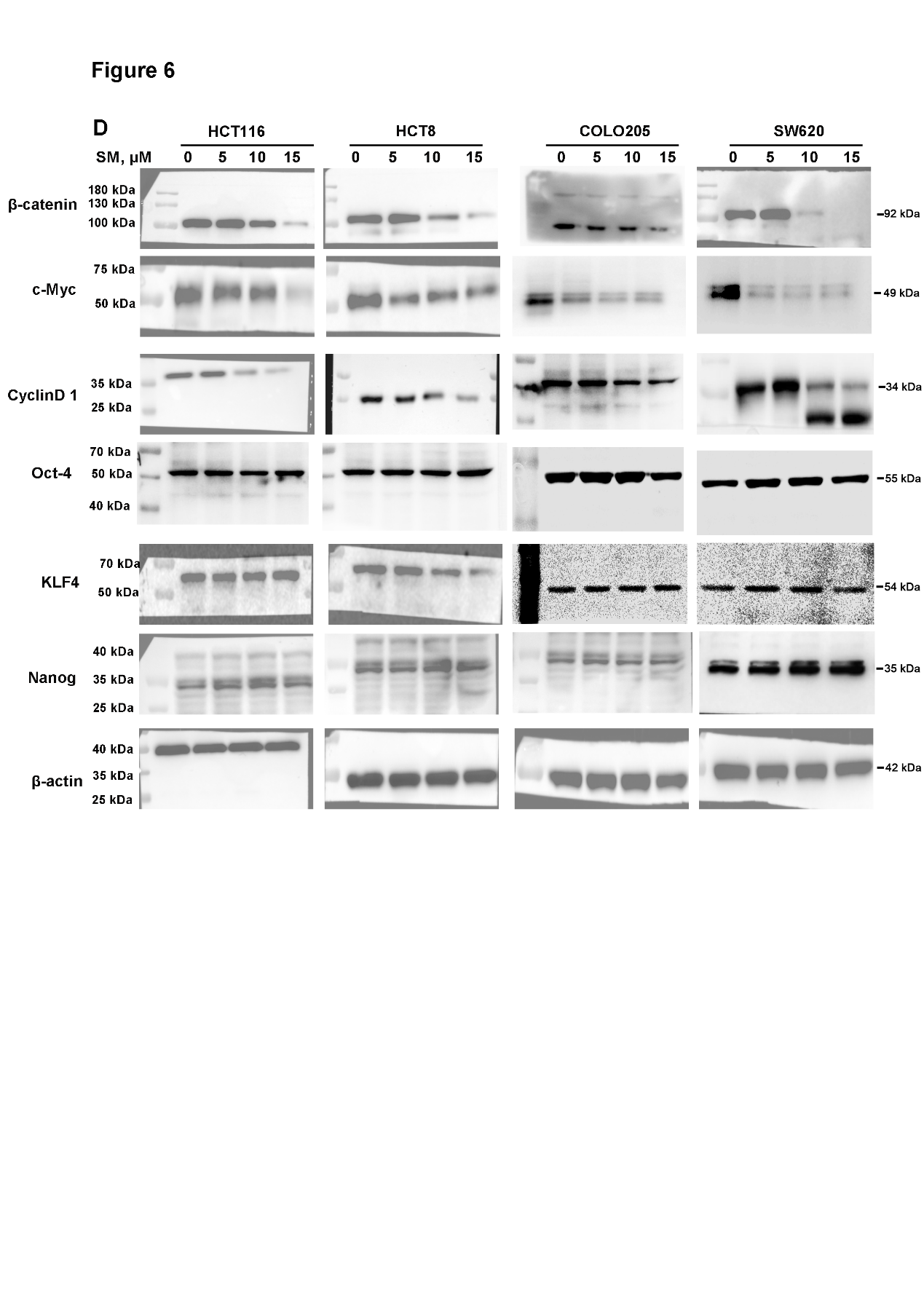


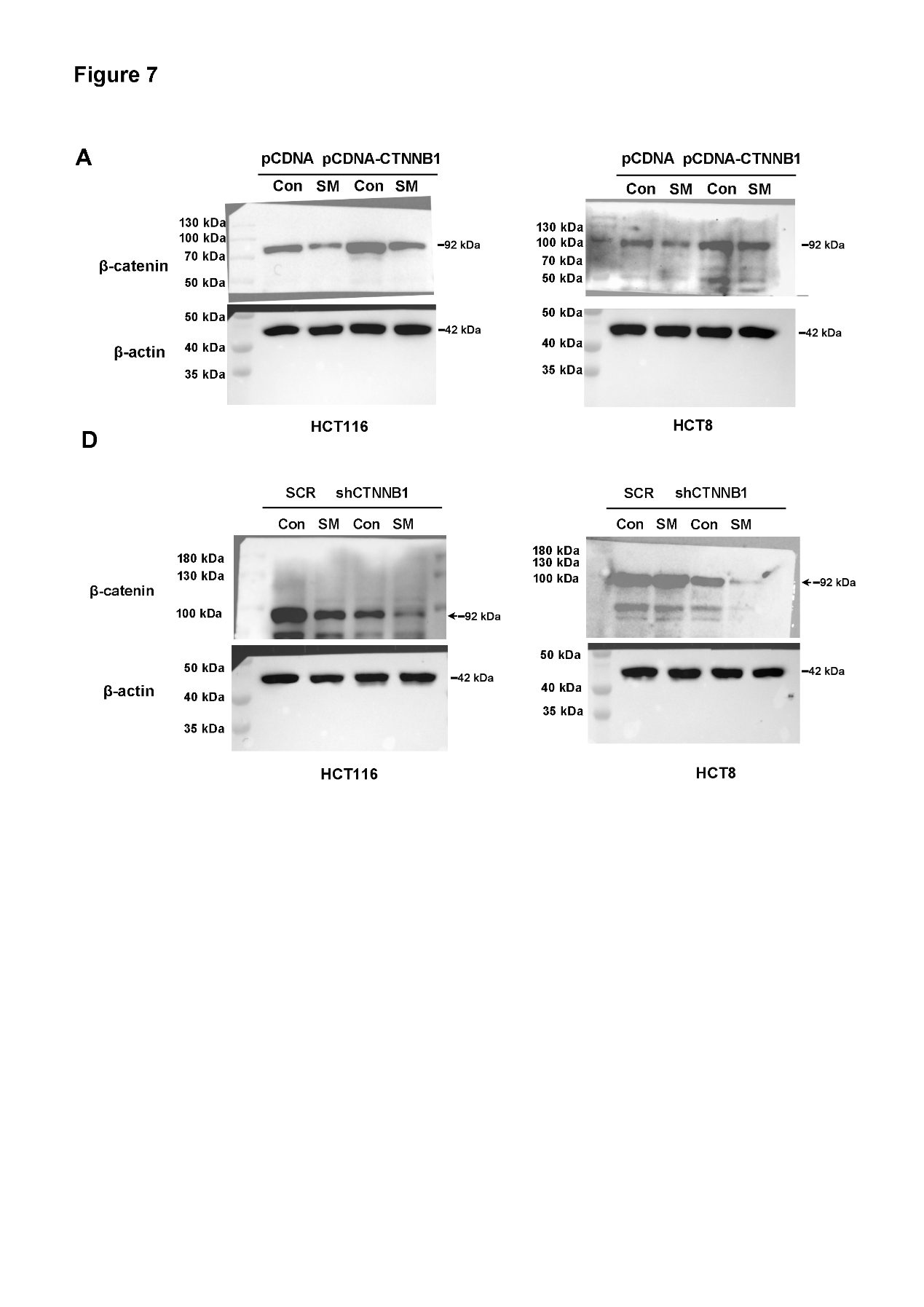


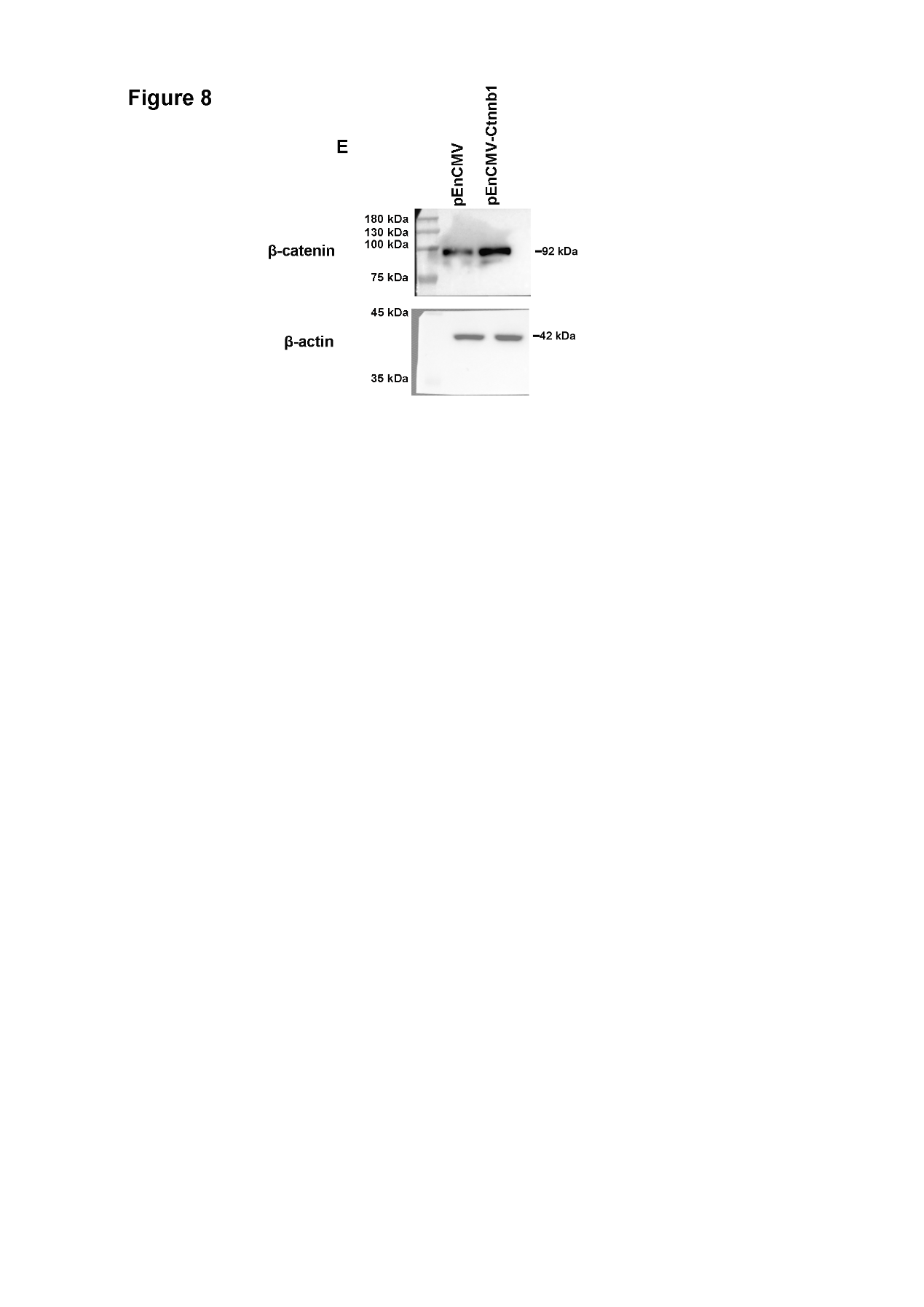


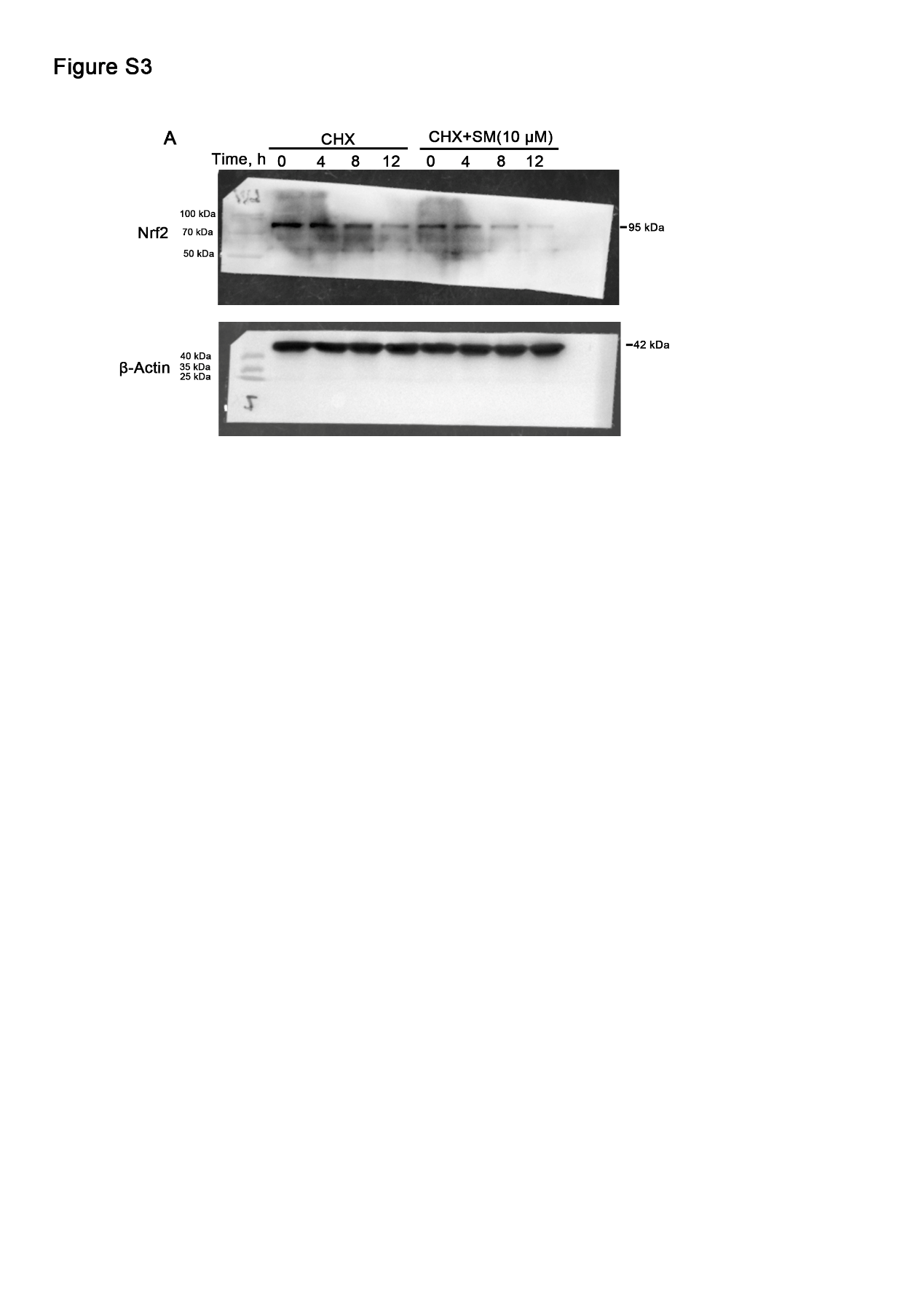


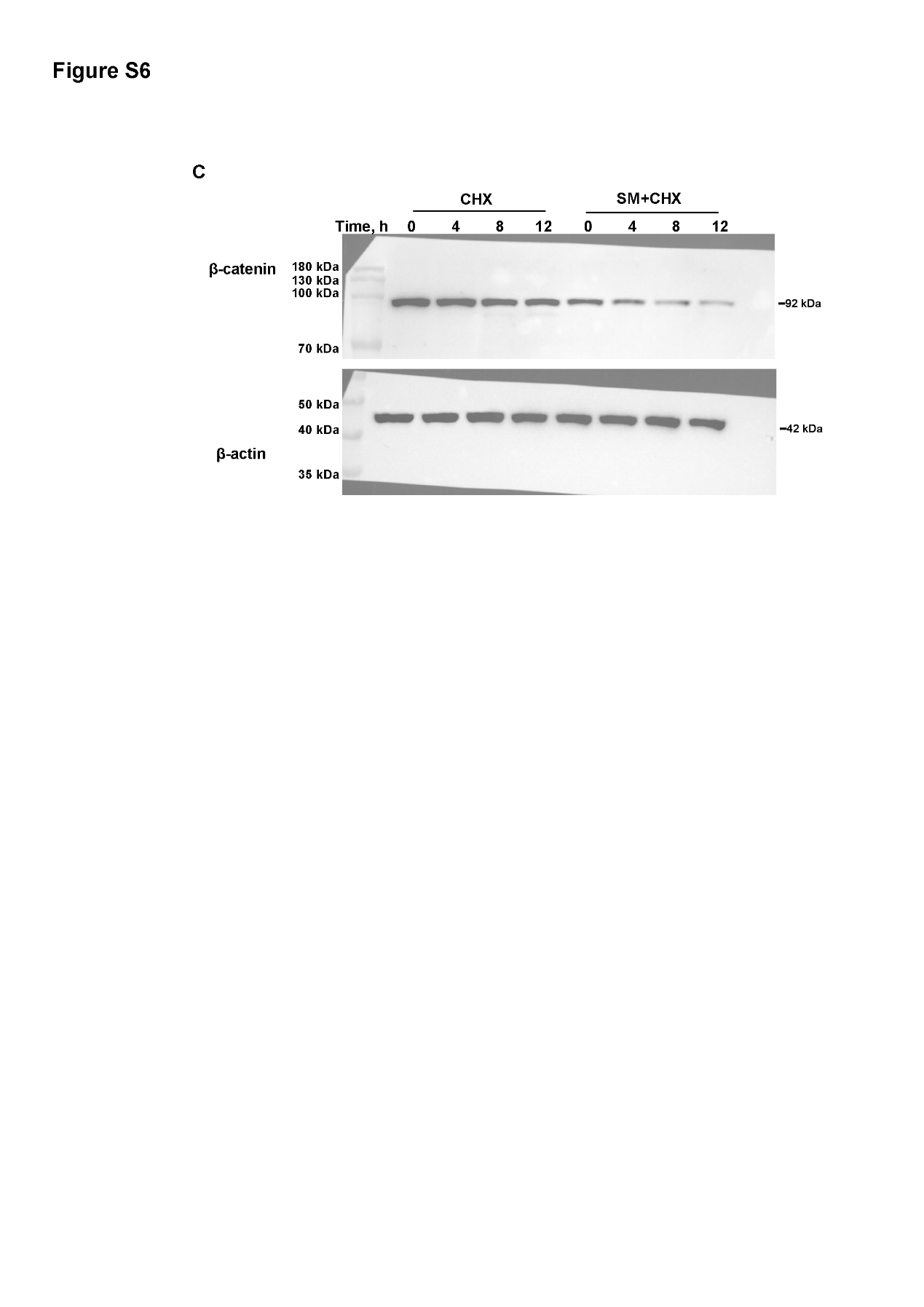

Supplement: Supplementary file 1 — Additional file 1 [file 13020_2025_1171_MOESM1_ESM.docx]
